# Evaluation of Protein Kinase Inhibitors with PLK4 Cross-Over Potential in a Pre-Clinical Model of Cancer

**DOI:** 10.3390/ijms20092112

**Published:** 2019-04-29

**Authors:** Amreena Suri, Anders W. Bailey, Maurício T. Tavares, Hendra Gunosewoyo, Connor P. Dyer, Alex T. Grupenmacher, David R. Piper, Robert A. Horton, Tadanori Tomita, Alan P. Kozikowski, Saktimayee M. Roy, Simone T. Sredni

**Affiliations:** 1 Division of Pediatric Neurosurgery, Ann and Robert H. Lurie Children’s Hospital of Chicago, Chicago, IL 60611, USA; aisuri@luriechildrens.org (A.S.); anbailey@luriechildrens.org (A.W.B.); cpdyer2@gmail.com (C.P.D.); ttomita@luriechildrens.org (T.T.); 2 Cancer Biology and Epigenomics Program, Stanley Manne Children’s Research Institute, Chicago, IL 60614, USA; 3 Department of Pharmacy, University of São Paulo, São Paulo, SP 05508-900, Brazil; maurício.tavares@usp.br; 4 School of Pharmacy and Biomedical Sciences, Curtin University, Bentley, Perth, WA 6102, Australia; hendra.gunosewoyo@curtin.edu.au; 5 Department of Ophtalmology, Universidade Federal de São Paulo, São Paulo, SP 04023-062, Brazil; alexgrups@gmail.com; 6 Thermo Fisher Scientific, Research and Development, Biosciences Division, Carlsbad, CA 92008, USA; David.Piper@thermofisher.com (D.R.P.); Robert.Horton@thermofisher.com (R.A.H.); 7 Department of Surgery, Northwestern University, Feinberg School of Medicine, Chicago, IL 60611, USA; 8 Star Wise Therapeutics, Madison, WI 53719, USA; alankozikowski@gmail.com; 9 Department of Pharmacology, Northwestern University, Feinberg School of Medicine, Chicago, IL 60611, USA; saktiroy069@gmail.com

**Keywords:** CFI-400945, CFI-400437, R1530, centrinone, axitinib, KW-2449, alisertib, AURK, rhabdoid tumor, AT/RT, medulloblastoma, protein kinase, brain exposure

## Abstract

Polo-like kinase 4 (PLK4) is a cell cycle-regulated protein kinase (PK) recruited at the centrosome in dividing cells. Its overexpression triggers centrosome amplification, which is associated with genetic instability and carcinogenesis. In previous work, we established that PLK4 is overexpressed in pediatric embryonal brain tumors (EBT). We also demonstrated that PLK4 inhibition exerted a cytostatic effect in EBT cells. Here, we examined an array of PK inhibitors (CFI-400945, CFI-400437, centrinone, centrinone-B, R-1530, axitinib, KW-2449, and alisertib) for their potential crossover to PLK4 by comparative structural docking and activity inhibition in multiple established embryonal tumor cell lines (MON, BT-12, BT-16, DAOY, D283). Our analyses demonstrated that: (1) CFI-400437 had the greatest impact overall, but similar to CFI-400945, it is not optimal for brain exposure. Also, their phenotypic anti-cancer impact may, in part, be a consequence of the inhibition of Aurora kinases (AURKs). (2) Centrinone and centrinone B are the most selective PLK4 inhibitors but they are the least likely to penetrate the brain. (3) KW-2449, R-1530 and axitinib are the ones predicted to have moderate-to-good brain penetration. In conclusion, a new selective PLK4 inhibitor with favorable physiochemical properties for optimal brain exposure can be beneficial for the treatment of EBT.

## 1. Introduction

Protein kinases (PKs) have the ability to transfer a γ-phosphate group from ATP to serine, threonine, or tyrosine residues. The human genome encodes 538 PKs. Most of them stimulate cell proliferation, survival, and migration, when constitutively overexpressed and, therefore, are associated with human cancer initiation and progression [1]. Targeted cancer therapies with small-molecule protein kinase inhibitors (PKI) have been developed to specifically block molecules that are either upregulated and overexpressed or mutated in tumor cells, thus minimizing toxicities, while improving treatment effectiveness [1,2]. PKI drugs represent a shift from the dominant cytotoxicity therapeutic approach to a signal transduction modulator (STM) approach. The STM approach, which is based on selective PKI drug candidates, offers the potential for an improved therapeutic safety index and a potential for better efficacy through treatment with selective PKI drug combinations to address the challenge of tumor heterogeneity.

Centrosomes are subcellular organelles, which form the bipolar spindle during mitosis through microtubule organization. Each centrosome has two centrioles embedded within a pericentriolar material, which contains critical proteins for microtubule nucleation as well as regulators of the cell cycle and its checkpoints. Centrioles are conserved microtubule-based organelles that form the core of the centrosome and have important roles in most microtubule-related processes, including motility, cell division, and cell signaling.

During mitosis, the two parental centrioles undergo centriole duplication to form centrosomes, which, after duplication, migrate to opposite poles of the cell, coordinating bipolar spindle formation for the perfect transmission of genetic material to the daughter cells [3]. Abnormalities in this process, such as the presence of three or more centrosomes in a cell (centrosome amplification), can result in genomic instability and aneuploidy, which have been shown to drive tumorigenesis [4,5,6,7]. Centrosome numbers are tightly controlled and coupled with DNA replication, cell cycle signaling, and progression. This regulation is key to protect genomic integrity [8].

Small molecules targeting centrosome components have been developed and tested in vitro and in vivo with some currently undergoing clinical trials [9]. The three main targets to centrosome control involve: (1) Centrosome duplication proteins (PLK1, CDK2, PLK4, AURKA), (2) centrosome amplification proteins (CHK1, PI3K, AKT, PLK4), and (3) centrosome clustering proteins (APC, HSP70, Stathmin) [9].

The polo-like kinase 4 (PLK4), a member of the polo-like kinase (PLK) family of the serine/threonine PKs, is a cell cycle-regulated protein recruited to the centrosome to promote the duplication of centrioles in dividing cells. Increase in PLK4 expression and kinase activity can lead to supernumerary centrioles, while PLK4 depletion can result in decreased centriole numbers [10]. Due to its critical role, in order to prevent centrosome amplification, PLK4 levels and activity must be strictly controlled.

All PLKs contain one or more Polo-box (PB) motifs. PB dimerization and binding to phosphoproteins regulate the activity of PKs by causing a change in its conformation. When aligned to PLK1, PLK4 has the lowest sequence homology (37%) of the catalytic domain, compared to PLK2 (53%) and PLK3 (54%), which influences substrate specificity [11]. While PLKs 1, 2, and 3 have two PB domains at their C-terminus, PLK4 possess three, which have important implications for its regulation and substrate selection [12,13]. Due to this characteristic, PLKs 1 through 3 bind to proteins that have previously been phosphorylated via their tandem PBs and form intramolecular heterodimers. Unlike the other PLKs, newly translated PLK4 is autoinhibited, which is quickly relieved by PB1-PB1- and PB2-PB2-mediated homodimerization of PLK4 monomers. Upon homodimerizing, both monomers of PLK4 are trans-autophosphorylated at Ser305, which generates a phosphodegron recognized by the ubiquitin ligase complex SCF/β-TRCP. Ubiquitination of PLK4 dissociates the homodimer and leads to the degradation of PLK4 [13,14,15,16,17,18].

PLK4 mRNA expression has been demonstrated to be comparatively low during quiescence, G0 phase, and early-to-mid-G1 phase, while gradually increasing in the late G1, S, and G2 phases, and peaking during mitosis. PLK4 protein becomes active only during S phase with the activity increasing to almost double in G2 phase [19].

Besides cancer, PLK4 aberrations also contribute to other human diseases. During development, autosomal recessive mutation of PLK4 leads to impairment of cell division through defects in the mitotic spindle. The resulting phenotype is one of delayed cell division, leading to microcephaly and primordial dwarfism in a Seckel syndrome spectrum. Furthermore, generalized retinopathy can occur with malformation of photoreceptor cells and apoptosis, probably due to disturbance of cilia development and retinal metabolic malfunction [20,21,22,23,24].

PLKs are of interest as therapeutic targets due to their druggability and a central role in cellular growth and proliferation. PLK4 overexpression and over-activation have been associated with a number of peripheral adult tumors like colorectal [25], breast [26,27], lung [28] cancer, melanoma [29], lymphoma [30], bone [31], gastric [32] and pancreatic [33] cancer; pediatric peripheral tumors like osteosarcoma [31], and adult central nervous system (CNS) tumors like glioblastoma multiforme (GBM) [34]. Paradoxically, downregulation of PLK4 has been discovered in liver cancer [35]. Recently, we reported PLK4 overexpression in pediatric embryonal tumors including peripheral malignant rhabdoid tumors (RT), atypical teratoid rhabdoid tumors (AT/RT) of the brain, medulloblastomas (MB), and neuroblastoma of the CNS (CNS-NB) [36,37,38,39].

Malignant RT (MRT) are rare, highly aggressive malignancies arising predominantly, but not exclusively, in infants and young children below the age of three years. They are commonly located in the CNS (65%) where they are called AT/RT. MRT can also be found in extracranial locations like the kidneys (9%) or other soft tissues (26%) including head and neck, liver, thorax, retroperitoneum, pelvis, and heart, among others [40,41]. AT/RTs are the most common malignant CNS tumors of children below six months of age [42,43]. Within registries (e.g., the Central Brain Tumor Registry of the United States, with 16,044 children registered from 2007–2011), AT/RTs account for approximately 40–50% of all embryonal CNS tumors in the first year of life [44]. Recently, integrated genomic, epigenetic, and clinicopathological data of large cohorts of patients’ samples have resulted in a better understanding of the clinical heterogeneity of these tumors and the identification of molecular subgroups with potential prognostic and therapeutic implications [45,46,47].

MB is the most common malignant brain tumor in children, accounting for nearly 20% of all childhood brain cancers and 40% of all childhood tumors in the posterior fossa. Similar to AT/RT, MBs are also embryonal in nature and consist of four distinct molecular subgroups: WNT, Sonic Hedgehog (SHH), group 3 (G3-MB), and group 4 (G4-MB). Each subgroup differs in demographics, transcriptomes, somatic genetic events, and clinical outcomes [48,49,50,51,52,53,54]. 

CFI-400945 is a multi-kinase inhibitor with relative selectivity for PLK4. It blocks PLK4 kinase activity and the associated aberrant mitoses [15,27,55]. We established that treatment with CFI-400945 impaired proliferation, survival, migration, and invasion as well as induced apoptosis, senescence, and polyploidy of MRT, AT/RT and MB tumor cells while sparing non-tumor human fibroblasts [36,37]. We also demonstrated synergy with classical DNA-damaging agents like doxorubicin and etoposide [36].

CFI-400945 is an optimized, indolinone-derived, selective ATP-competitive kinase inhibitor with nanomolar affinity for PLK4 [55]. While displaying minimal inhibition of the other members of the PLK family, CFI-400945 has been shown to inhibit members of the Aurora kinase (AURK) and tropomyosin receptor kinase (TRK) families but requiring concentrations in order of magnitude higher than PLK4 [55]. CFI-400945 has been shown to be efficacious in decreasing tumor size in vivo in xenograft models of colon [56], pancreatic [33], breast [27], lung [28], and AT/RT [36]. This small molecule inhibitor is currently being tested in clinical trials for breast (NCT03624543), prostate (NCT03385655), advance adult solid tumors (NCT01954316), and acute myeloid leukemia/myelodysplastic syndromes (NCT03187288).

Besides CFI-400945, a handful of PKIs with PLK4 crossover potential and anticancer activity have been described in the literature including: R1530 [57,58], centrinone/centrinone B [59], CFI-400437 [60], axitinib [60,61,62], KW-2449, [63,64], and alisertib [65,66].

CFI-400437 is an indolinone-derived, ATP-competitive kinase inhibitor [60] with high selectivity for PLK4, but also displays lower levels of inhibitory activity for the other members of the PLK family. CFI-400437 was found to significantly impair the growth of breast cancer cells in vitro, as well as decreasing tumor size in mouse xenograft tumor models [60]. 

Centrinone and centrinone B are selective, reversible PLK4 inhibitors (PLK4i) developed using the pan-AURK inhibitor VX-680 as a template. Centrinone reversibly depleted centrioles by preventing centriole assembly in HeLa cells resulting in a p53-dependent cell cycle arrest in G1 phase. Centrinone B exhibited comparable outcomes with centrinone [59].

R1530 is a member of a series of pyrazolobenzodiazepine compounds, which were identified as mitotic inhibitors. R1530 is a multi-kinase inhibitor which was shown to target all the five members of the PLK family. It induced polyploidy that led to apoptosis of cancer cells by interfering with the mitotic checkpoint kinase BubR1, which is likely to be a result of its PLK4 inhibition [58]. 

Axitinib was developed as a tyrosine kinase inhibitor, specifically with high affinity for VEGFR1–3 [67]. Prior to FDA approval, axitinib was found to inhibit PLK4 with an IC_50_ value of 4.2 nM [67]. Axitinib was used during the synthesis of CFI-400437, as a model of a potent PLK4i [60]. In 2012, axitinib was approved by the FDA for use in renal cell carcinoma [57,68,69] and has since been in clinical trials to treat thyroid [70] and advanced non-small cell lung [71] cancer as well as melanoma [61,72].

KW-2449 is an analog of CFI-400945 that we identified in an attempt to find a candidate molecule with potentially better brain exposure based on its chemical properties. It has been previously described as a multi-kinase inhibitor of FLT3, ABL, ABL-T315I, and AURKs. Potent inhibitory growth effects on leukemia cells with FLT3 mutations were achieved by inhibition of the FLT3 kinase, with subsequent down-regulation of phosphorylated-FLT3/STAT5, G1 arrest, and apoptosis [63,64]. 

Alisertib is a pyrimidobenzazepine, ATP competitive inhibitor for the AURK family with the highest selectivity for Aurora kinase A (AURKA) [73]. AURKA has previously been described to be overexpressed in AT/RT [74] and alisertib is currently in a phase II clinical trial for its treatment (NCT02114229).

It is estimated that >95% of approved drugs lack sufficient blood-brain-barrier (BBB) penetration to allow efficacy [75,76,77]. The tissue exposure challenge is even greater for PKI drugs [78]. Briefly, an initial informatics analysis of differences in molecular properties between approved CNS drugs and PKI drugs approved or in public domain clinical trials databases identified a cluster of three molecular properties that distinguished PKIs from approved CNS drugs. CNS drugs clustered around a multiproperty profile of molecular weight (MW ≤ 400), lipophilicity (LogP ≤ 4), and polar surface area (PSA ≤ 80), with extant PKIs often falling outside this multi-property profile, especially PSA. This pioneering observation was updated and extended to undisclosed internal CNS candidates within Pfizer as well approved CNS drugs [79,80]. The investigators proposed a CNS multiproperty profile, based on analysis of their proprietary database, that could be used for late stage medicinal chemistry optimization [79]. Continuing trends in multi-property consideration for CNS drug candidate design and refinement are now embedded in commercially available computational packages such as ACD Labs Percepta as a virtual screen for early alerts related to potential brain penetration.

Herein, we evaluate multiple small molecule PKI with potential PLK4 crossover for their effects in a pre-clinical cellular model of EBT, potential to occupy the PLK4 active site in virtual docking analyses, and their potential liability for BBB penetration based on computed multi-property profile.

**Figure 1:**
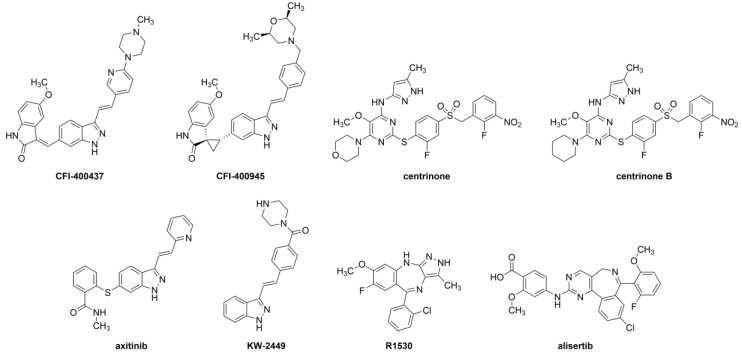
Chemical structures of the evaluated protein kinase inhibitors.

Our aim was to evaluate the abovementioned inhibitors (Figure 1) in a pre-clinical model of embryonal tumors (RT and MB) in regard to their anti-cancer properties and potential for brain exposure. The predicted binding modes of these inhibitors were also examined, with the goal of identifying the structural requirements for potential to binding to the PLK4 active site.

## 2. Results

### 2.1. Structural Analysis of the Inhibitors 

#### 2.1.1. Docking Simulations

**Figure 2:**
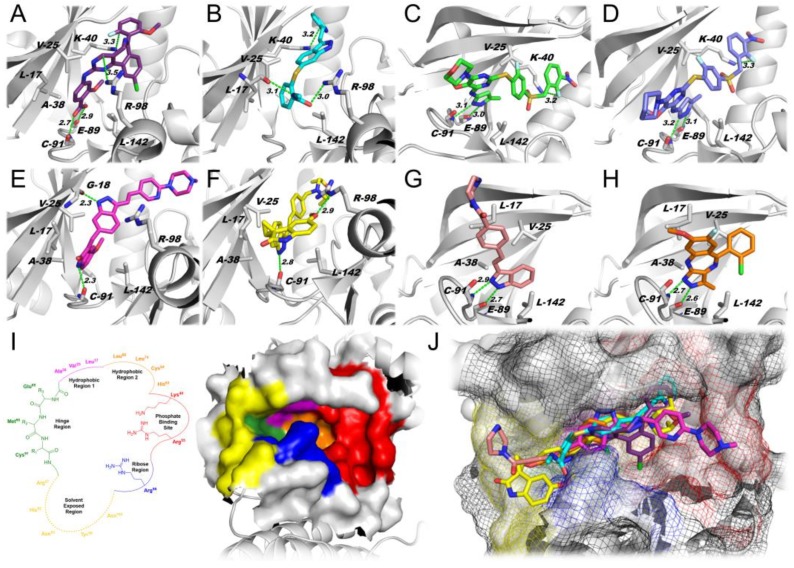
PLK4 *in silico* findings. (**A**) Docking pose of alisertib (carbons are depicted in purple). (**B**) Docking pose of axitinib (carbons are depicted in cyan). (**C**) Empirical complex of centrinone bound to PLK4 (Protein Data Bank (PDB) entry: 4YUR, carbons are depicted in green). (**D**) Docking pose of centrinone B (carbons are depicted in navy blue). (**E**) Docking pose of CFI-400437 (carbons are depicted in magenta). (**F**) Docking pose of CFI-400945 (carbons are depicted in yellow). (**G**) Docking pose of KW-2449 (carbons are depicted in light pink). (**H**) Docking pose of R1530 (carbons are depicted in orange). (**I**) Two-dimensional (2D, left) and three-dimensional (3D, right) schematic representation of the ATP-binding pocket of PLK4. (**J**) Superimposed pose of the inhibitors at the PLK4 binding cavity. Cartoon protein depicted in white. Carbons of PLK4 are depicted in white. Oxygen is depicted in red. Nitrogen is depicted in blue. In panels C and D, sulfur is depicted in yellow. In panels A, C, D, and H, fluorine is depicted in cyan. In panels A and H, chlorine is depicted in green. Hydrogen bonds are indicated as green dashed lines. Interatomic distances in angstroms (Å). A: alanine; C: cysteine; E: glutamate; G: glycine; K: lysine; L: leucine; V: valine; R: arginine.

Docking simulations using the PLK4 catalytic cavity and each of the various PKIs with PLK4 crossover activity (Figure 1) demonstrated that all of the tested inhibitors, except axitinib, exhibited similar interactions within the binding cavity, engaging in H-bonding with backbone residues Glu-89 and Cys-91 (Figure 2A–H). Only axitinib did not engage in H-bonds at the hinge region (Figure 2I) with Glu-89 and Cys-91. Instead, axitinib exhibited two H-bonds with the backbone of Leu-17 and the side chain of Arg-98 at the ribose pocket (Figure 2I). Interestingly, alisertib (Figure 2A) and axitinib (Figure 2B) engaged in an additional cation-π interaction with Lys-40, while the empirical structure of centrinone [59] (Figure 2C) and the docking pose of centrinone B (Figure 2D) both engaged in cation–dipole interactions with this residue (−NH_3_^+^ F). These findings may explain the higher docking scores obtained for these inhibitors (Table 1). Compounds CFI-400437 and CFI-400945 displayed extra H-bonding with Gly-18 and Arg-98, respectively, and a similar exploration of the phosphate-binding site (Figure 2E,F,I,J). Compounds KW-2449 and R1530 only exhibited polar interactions at the hinge region that may explain their lower scores as presented in Table 1. Noteworthy, the docking findings also highlighted the potential of KW-2449 and R1530 to guide further structure–activity relationship (SAR) studies as well as the design of novel compounds with improved exploration of the PLK4 binding cavity.

Additional hydrophobic interactions have also been observed through the cavity for all inhibitors especially—but not exclusively—with Leu-17, Val-25, Ala-38, and Leu-142 (Figure 2A–H). The overlapping poses of the compounds (Figure 2J) indicated a similar exploitation of the binding cavity by the eight compounds examined. Therefore, compounds bearing polar groups like piperazine (CFI-400437 and KW-2449) and morpholine (centrinone and CFI-400945) had a better fit within the hydrophilic regions of the pocket (i.e., phosphate-binding site and ribose pocket, Figure 2I,J), or turned their polar moieties to the solvent-exposed region.

#### 2.1.2. Physical Property Analysis

The logBB is a logarithm index that express the brain/plasma concentration ratio of drugs, and it is indicative of the permeation of drugs through the BBB. The logBB values from ACD Labs Percepta 2016 (Build 2911) identify differences among the inhibitors, including those with a common indazole core (Table 2). 

In this regard, the CFI-400945 profile is: MW = 534.65, PSA = 79.48, and calculated LogP (cLogP) = 5.05, which puts CFI-400945 at the cusp of multiple property profiles for drugs with good brain exposure. Similarly, other tested compounds had one or more of the multi-property computed values: Centrinone (MW = 633.65, PSA = 204.84, cLogP = 4.03); centrinone B (MW = 631.68, PSA = 195.61, cLogP = 4.31); and alisertib (MW = 518.92, PSA = 105.93, cLogP = 5.82). Inhibitors with profiles that forecast less risk for BBB penetrance include: CFI-400437 (MW = 492.57, PSA = 86.38, cLogP = 4.07); R1530 (MW = 356.78, PSA = 62.30, cLogP = 3.82); KW-2449 (MW 332.40, PSA =61.02, cLogP = 2.56); axitinib (MW = 386.47, PSA = 95.97, cLogP = 3.65). 

Overall, centrinone and centrinone B represent those with higher computed risk based on total polar surface area (TPSA) > 190 Å^2^ as well as > 600 MW. Clearly, future studies for brain tumor treatment research warrant an exploration of dose-dependent brain exposure in order to provide a firm experimental foundation.

### 2.2. Kinase Assays 

**Figure 3:**
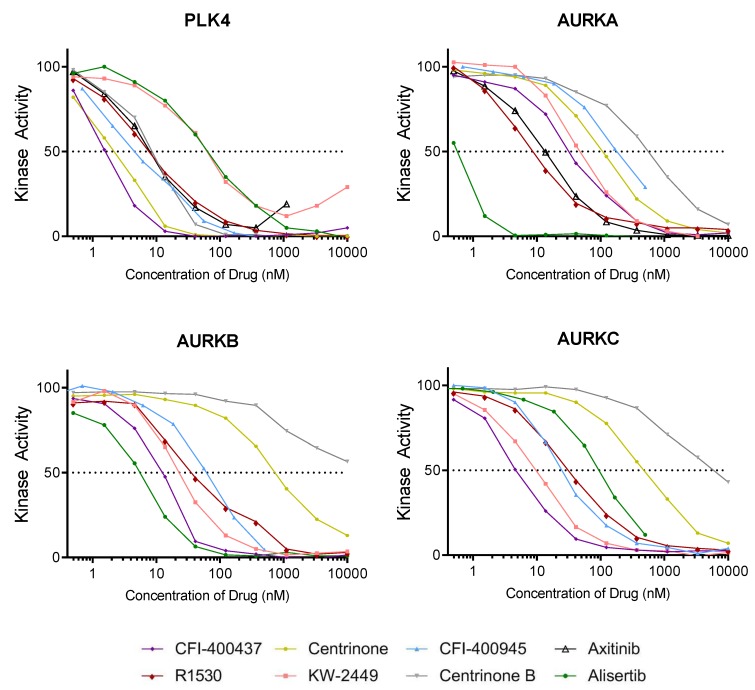
Kinase activity curves for PLK4, Aurora kinase A (AURKA), Aurora kinase B (AURKB), and Aurora kinase C (AURKC) in the presence of various concentrations of for each inhibitor. (SelectScreen™ Kinase Profiling Services—Thermo Fisher Scientific, Carlsbad, CA, USA).

Kinase assays were performed to determine the half-maximum inhibitory concentrations (IC_50_) for PLK4. Because PLK4 has high catalytic similarities with AURKs [60,81,82], the IC_50_ for AURKA, Aurora kinase B (AURKB), and Aurora kinase C (AURKC) were also determined for each of the inhibitors included in this study. Results shown in Figure 3 and Table 3 indicate the following: (1) CFI-400437 had the lowest IC_50_ value for PLK4 (1.55 nM) followed by centrinone (2.71 nM) and CFI-400945 (4.85 nM); (2) centrinone and centrinone B were the most specific to PLK4; (3) CFI-400437 inhibited both AURKB and AURKC at concentrations <15 nM, while CFI-400945 inhibited AURKB at a higher concentration (70.7 nM) and AURKs A and C at even higher concentrations (188 nM and 106 nM, respectively); (4) high IC_50_ for PLK4 was observed for KW-2449 (52.6 nM) which inhibited all three AURKs more efficiently than PLK4; (5) finally and not surprisingly, alisertib, which is known to be a primary inhibitor of AURKA, had the highest IC_50_ for PLK4 (62.7 nM) with the best inhibitory activity of all AURKs. 

### 2.3. Cell-Based Studies

**Figure 4:**
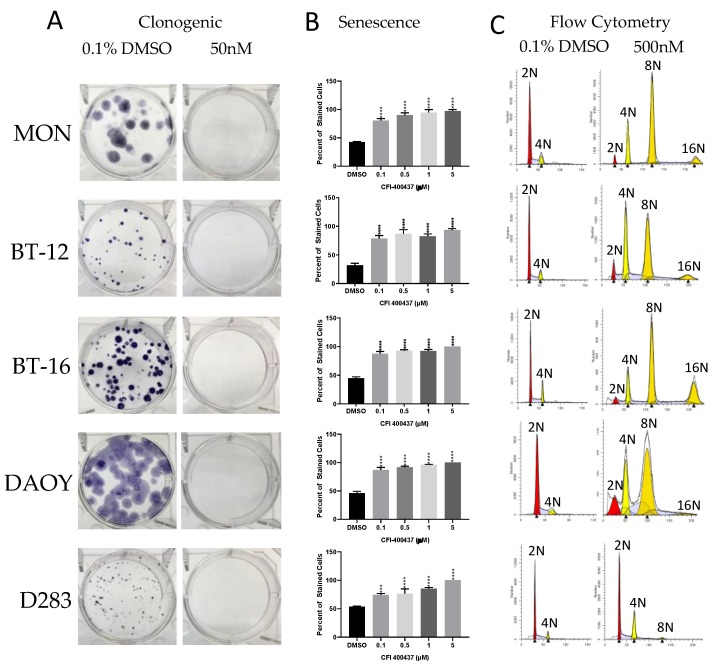
Phenotypic evaluation of CFI-400437 in multiple cells lines. (**A**) Clonogenic - colony formation assay of RT and MB cells treated with 50nM CFI-400437 reveals complete inhibition of colony formation in all cell lines. (**B**) Beta-galactosidase assay shows induction of cell senescence when treated with CFI-400437 in all cell lines (*** *p* < 0.001 and **** *p* < 0.0001, one-way ANOVA). (**C**) Cell cycle analysis reveals the induction of polyploidy with 500 nM CFI-400437.

**Figure 5:**
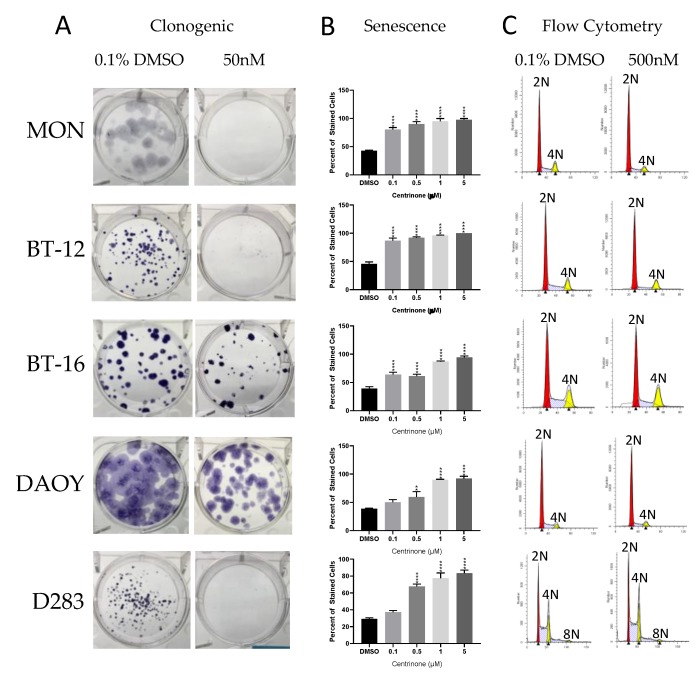
Phenotypic evaluation by centrinone inhibition in multiple cells lines. (**A**) Clonogenic - colony formation assay of RT and MB cells treated with 50nM centrinone reveals complete inhibition of colony formation in all cell lines except the MB cell line DAOY and the RT cell line BT-16. (**B**) Beta-galactosidase assay shows induction of cell senescence when treated with centrinone in all cell lines (** *p* < 0.01, *** *p* < 0.001 and **** *p* < 0.0001, one-way ANOVA). (**C**) Cell cycle analysis reveals no polyploidy when treated with 1µM centrinone in all cell lines.

**Figure 6:**
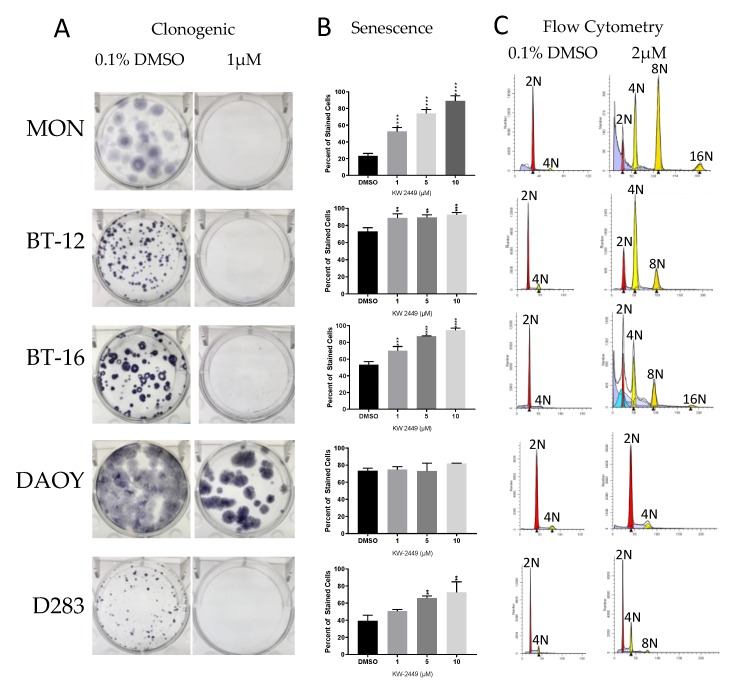
Phenotypic evaluation by KW-2449 in multiple cells lines. (**A**) Clonogenic - colony formation assay of RT and MB cells treated with 1µM KW-2449 reveals complete inhibition of colony formation in all cell lines except the MB cell line DAOY. (**B**) Beta-galactosidase assay shows a dose-dependent induction of cell senescence when treated with increasing doses of KW-2449 in all cell lines (** *p* < 0.01, *** *p* < 0.001 and **** *p* < 0.0001, one-way ANOVA). (**C**) Cell cycle analysis reveals the induction of polyploidy with 2µM KW-2449 in all cell lines except DAOY.

The evaluation of the impact of each inhibitor over cell phenotype (proliferation, viability, colony formation, senescence, and cell cycle/polyploidy) demonstrated that: (1) Decrease in viability and proliferation was consistent with the results of the kinase assay (Figure 3 and Table 3) being more significant for CFI-400437 and centrinone (Table 4, Figure 4 and Figure 5), followed by CFI-400945, which has been previously tested in our system [36,37]; (2) although KW-2449 had the second highest IC_50_ on the kinase assay, significant impact over viability/proliferation, inhibition of colony formation, induction of senescence and polyploidy were observed, but in higher concentrations (1–2 µM) (Figure 6); (3) centrinone, which is the most selective inhibitor for PLK4, together with centrinone B, did not induce polyploidy in the cell lines tested (Figure 5). AURKB proper function during mitosis is crucial for ensuring the prevention of mitotic errors, endoreduplication, and polyploidy [83,84,85]. Therefore, this phenomenon could stem from the absence of AURKB-associated inhibition, which may be necessary for induction of polyploidy [86].

### 2.4. KW-2449

Although kinase assays reveal this ligand to possess a high IC_50_ for PLK4 and cell-based assays indicated a phenotype consistent with PLK4 inhibition at high concentrations, the docking simulations indicated that KW-2449 has the potential to guide further structure–activity relationship (SAR) studies and the design of novel compounds with improved exploration of the PLK4 binding cavity. For this reason, and because of the favorable scores for BBB penetration, we further studied KW-2449.

#### 2.4.1. KW-2449 Potential for Cardio Toxicity and Drug-Drug Interaction Risk

Screening of a PKI for human ether-a-go-go-related gene (hERG) potassium channel inhibition has become an early step in testing for potential drug dependent long QT syndrome that is linked to sudden death [87]. Binding of KW-2449 at the hERG channel was not detected under standard assay procedures at the highest concentration tested (IC_50_ > 10 µM) (Supplementary Figure S1). These data suggest that KW-2449 is not in the high-risk category for cardiovascular toxicity. Similarly, cytochrome P450 (CYP450) enzymes, which are drivers of first-pass metabolism for orally administered drugs, can contribute to toxicities or therapeutic failures [88] with the two of the most significant in risk being CYP3A4 and CYP2D6, which showed an IC_50_ value >10 µM (the highest tested concentration) (Supplementary Figure S1). 

#### 2.4.2. KW-2449 Kinase Inhibition Screen

We performed a kinase selectivity screen and established that KW-2449 has low selectivity, inhibiting PLK4 among several other kinases. The screening revealed that 215 out of 486 (44%) kinases had their activity inhibited above 80% by KW-2449 at 10 µM (Supplementary Table S1). The IC_50_ for selected kinases was validated by a 10-point titration curve analysis. The IC_50_ values of AURKA, AURKB, and AURKC were 45.8 nM, 23.8 nM, and 23.2 nM, respectively while the IC_50_ value of PLK4 was even higher: 52.2 nM (Figure 3 and Table 3). In summary, KW-2449 showed low selectivity and efficient PLK4 inhibitory effect inducing a phenotype consistent with PLK4 inhibition in EBT cells. Key features suggest a potential for low cardiotoxicity or drug–drug interaction risk.

## 3. Discussion

PKIs have promised to overcome major disadvantages of traditional cancer treatments as they potentially discriminate between non-malignant and rapidly proliferating cancer cells, leading to fewer off-target effects and lower toxicity for the patients. We previously demonstrated overexpression of PLK4 in embryonal tumors including RT, pediatric MB, and CNS-NB, for which only highly toxic and poorly effective treatments are available. Our preliminary findings suggested that targeting PLK4 with small-molecule inhibitors may represent a novel strategy to treat EBT and possibly other tumors of the brain [36,37,38]. We have demonstrated that oral CFI-400945 delayed tumor growth and improved survival in a xenograft model of AT/RT [35]. However, CFI-400945 is at the cusp of the multi-property profile predictive of efficient brain exposure and therefore, this favorable outcome may be a result of the disruption of the BBB by the xenografting procedures, rather than the ability of the molecule to penetrate the brain. This type of BBB disruption can be responsible for over-predicting the efficacy of agents that otherwise would not penetrate the brain. This explanation is one of the reasons why certain agents that were shown to be active in preclinical studies failed in clinical trials [89].

A brain penetrant PLK4i could be used to treat not only EBT but other aggressive CNS tumors with PLK4 overexpression like GBM [34]. Therefore, we conducted the present study in order to understand the potential of available PLK4is and explore the possibility to develop new chemical entities with higher PLK4 selectivity and efficient BBB penetration.

### 3.1. Polyploidy and Aurora Kinases (AURKs)

Polyploid cells have more than two paired homologous chromosomes. Aberration of the homeostatic diploid state, resulting in polyploidy, has been implicated in a number of cancers [90,91,92,93], but it can also increase cancer cells’ susceptibility to cytotoxic therapies [94]. Cell fusion, cytokinesis failure, and endoreduplication are three mechanisms that can lead to polyploidization [92]. 

It has been demonstrated that the well-balanced action of the PLK and AURK families of protein kinases is essential for normal mitotic function. AURKs are members of a conserved family of serine/threonine PKs involved in cell cycle progression [85]. AURKA is involved in organizing cell polarity, regulating the assembly and organization of mitotic spindles, centrosome maturation, and regulation of mitotic checkpoints [81,95]. AURKB is actively involved in mitosis, chromosomal alignment and segregation, formation of the cleavage furrow, and control of checkpoints, and therefore prevents the occurrence of aneuploidy [83,85,86]. In fact, inhibition of AURKB has been found to lead to polyploidy in vitro in both cancer and non-cancer cells [83,84,86,96]. AURKC is the least-studied member of the family. It is known to be highly expressed in testis and involved in the coordination of meiotic spindles during spermatogenesis [81,82]. While the active sites of AURKB and AURKC are identical, the active site of AURKA differs by only three amino acids [82]. Whereas inhibitors specific to AURKB and pan-AURK inhibitors induced significant polyploidy in vitro, the phenotype was not observed when subjected to inhibition by an AURKA selective inhibitor [84]. 

PLK4 shares 37% residue homology with that of AURKA, making the active site of PLK4 more structurally similar to the members of the AURK family than to PLK1 [60,72,81,82]. This high degree of homology between the ATP-dependent catalytic domains could explain the observed inhibition of AURKs when subjected to PLK4is. Because of these catalytic similarities between PLK4 and AURKs, we evaluated the kinase inhibitory activity of all three AURKs for each PLK4i.

Previously, we have observed induction of polyploidy after treating EBT cells with CFI-400945 [36,37]. We interpreted this outcome as a result of DNA endoreduplication in the absence of centriole replication, which would have been resultant from PLK4 inhibition. However, our results showed that centrinone, the most selective inhibitor for PLK4, did not induce polyploidy of the cancer cells (Figure 3B), which raises the hypothesis that the major factor responsible for polyploidy is inhibition of AURKB rather than PLK4. 

Although the majority of drug developing efforts have focused on selectivity for a single biological target, there has been evidences implying that more selective drugs are less likely to work in the clinic compared with drugs that target a set of molecular pathways implicated in the disease pathophysiology [77]. In this scenario, aiming for a PKI targeting multiple desirable targets may be of greater interest. 

### 3.2. Considerations for Future Development of a Brain Penetrant PLK4i and its Therapeutic Use

In order to identify improved inhibitors for use in the treatment of brain cancers, it will be important to design compounds that are not only selective for PLK4, but that also have appropriate lipophilicity (logP) and as well as modest total polar surface areas (TPSA) to allow for brain penetration. Thus, the use of multi-method predictive modules such as central nervous system multi-parameter optimization (CNS MPO) should be considered in the compound optimization process. KW-2449 and R1530 appear to have reasonable scores for brain exposure while centrinone and centrinone B are the most selective in terms of their kinase profiles. These results together with further molecular docking studies may lead to the design of compounds with improved selectivity and brain penetration, thus allowing for improved cancer therapeutics.

Kinase inhibitors can be used in combination with cytotoxic chemotherapy, radiation therapy, or other STMs. In previous studies, we established the synergism of the PLK4i CFI-400945 with cytotoxic chemotherapy drugs [35]. A key challenge in the clinics is to identify the most efficient combination of kinase targets and then develop treatment combinations for specific cancer types. Since the PLK4 inhibition has a well-defined mechanism of action, combination with drugs that act on cooperating pathways are potentially significant. For example, Kawakami et al. [28] found that combined treatments with CFI-400945 and seliciclib, a CDK2 inhibitor, exerted both additive and synergistic effects to reduce lung cancer cell growth. Therefore, combining a PLK4i not only with cytotoxic drugs but also with drugs that affect cooperating pathways may be promising.

## 4. Materials and Methods

### 4.1. Inhibitors Tested

We studied 8 PKI with PLK4 cross-over potential: CFI-400945 (CAS#1338800-06-8, Cat#16850) (Cayman Chemical, Ann Arbor, MI, USA) [55]; CFI-400437 (CAS#1247000-76-5, Cat# SYN-1207) (SynKinase, San Diego, CA, USA) [60]; centrinone (CAS#1798871-30-3, Ludwig Institute for Cancer Research, New York, NY, USA) [59]; centrinone B (CAS#1798871-31-4, Ludwig Institute for Cancer Research, New York, NY, USA) [59]; KW-2449 (CAS# 1000669-72-6, Cat#HY-10339) (MedChemExpress, Monmouth Junction, NJ, USA) [63]; R1530 (CAS#882531-87-5, Cat#15225) (Cayman Chemical, Ann Arbor, MI, USA) [57]; axitinib (CAS#319460-85-0, Cat#13813) (Cayman Chemical, Ann Arbor, MI, USA) [61], and alisertib (CAS#1028486-01-2, Cat# HY-10971) (MedChemExpress, Monmouth Junction, NJ, USA) [97] (Figure 1).

### 4.2. Structural Analysis of the Inhibitors

The crystallographic structure of PLK4 was obtained from the Protein Data Bank (PDB) with access code 3COK (https://www.rcsb.org/structure/3cok). This structure has a resolution of 2.25 Å. All the inhibitors’ structures were built and energy-minimized with the density functional theory (DFT) method Becke-3-Lee Yang Parr (B3LYP) with the standard 6-31G* basis set available in the Spartan’14 program (Wavefunction, Inc., Irvine, CA, USA). Molecular docking was performed with the GOLD 5.2 program (CCDC). The scoring function used was ‘GoldScore’, which is the default function of the GOLD 5.2 program. Hydrogen atoms were added to PLK4 according to the data inferred by the GOLD 5.2 program on the ionization and tautomeric states. The docking interaction cavity in the protein was established with a 10 Å radius from the co-crystalized ligand (phosphoaminophosphonic acid-adenylate ester). The number of genetic operations (crossover, migration, mutation) in each docking run that was used in the search procedure was set to 100,000. Docking simulations were performed six times for each inhibitor. The figure of the best—and most frequent—docking pose for each compound was generated by the PyMOL Delano Scientific LLC program (Palo Alto, CA, USA). The experimental structure of centrinone bond to PLK4 was obtained from the PDB with the access code 4YUR. CNS MPO scores were calculated as per the original method described by Pfizer. Calculated logP, log D, TPSA, MW, pKa, and HBD were obtained using ACD Labs Percepta 2016 (Build 2911). The logBB values were obtained using ACD Labs Percepta software v16 (Advanced Chemistry Development, Inc., Toronto, On, Canada).

### 4.3. Kinase Assay

Each inhibitor’s potency was determined by generating 10-point IC_50_ curves from a 4-fold dilution series in DMSO (1 mM). Curves were generated from the compound concentration and the corresponding percent (%) inhibition calculated for each concentration tested. For PLK4, we used the LanthaScreen Eu Kinase Binding Assay (Thermo Fisher Scientific, Carlsbad, CA, USA) that utilizes an Alexa Fluor conjugated “tracer” and an Eu-labeled anti-tag antibody to measure binding of a compound to the kinase target. For AURKs (AURKA, AURKB and AURKC) we used the Z’ LYTE Kinase Assay (Thermo Fisher Scientific, Carlsbad, CA, USA) that determines the differential sensitivity of phosphorylated and non-phosphorylated peptide substrates to proteolytic cleavage using a FRET-based readout.

### 4.4. Cell Culture

Six well-established and extensively characterized cell lines were used in this study: The rhabdoid cells MON provided by Dr. Delattre (Institute Curie, Paris, France) [98], the AT/RT cell lines BT-12 and BT-16, which have been extensively used in preclinical studies [99], and two MB cell lines (DAOY and D2830). The cells were cultured as previously described.

### 4.5. Cell-Based Assays

For each cell line and each inhibitor, we performed proliferation, viability, senescence, colony formation, and cell cycle analysis, as described below.

#### 4.5.1. Proliferation Assay

To evaluate cell proliferation, the TACS MTT Cell Proliferation Assays (Trevigen, Gaithersburg, MD, USA) were used. The RT cell lines (MON, BT-12, and BT-16) were plated at 2 × 10³ cells on each well of a 96-well plate. The MB cell lines (DAOY and D283) were plated at 5 × 10^4^ and 5 × 10^5^ cells per well, respectively. The absorbance was measured after 24, 48, 72, and 96 h at concentrations ranging from 0.001 to 10 µM of the inhibitor. All assays were performed in triplicates.

#### 4.5.2. Viability Assay

Cell viability was assessed using the Presto Blue™ Cell Viability reagent (Thermo Fisher Scientific, Carlsbad, CA, USA) following the manufacturer’s instructions. Cells were plated in 96-well plates at the same densities described above for the proliferation assay. The fluorescence was measured after 24, 48, 72, and 96 h at concentrations ranging from 0.001 to 10 µM of the inhibitor. All assays were performed in triplicate.

#### 4.5.3. Clonogenic Assay

All cell lines were seeded at 200 cells per well in six-well plates and incubated overnight. After 24 h, the treatment medium was added and changed once a week. Cells were treated with 0.01, 0.05, 0.1, 0.2, 0.5, 1, 5, and 10 µM of PLK4i and 0.1% DMSO as control. After 14 days, the cells were washed twice with 1x PBS, fixed with formalin, and stained with Cresyl violet (ACROS Organics, Pittsburgh, PA, USA). Colonies were counted using the ImageJ software (www.imagej.nih.gov, accessed on April 21, 2017).

#### 4.5.4. Beta-Galactosidase Assay

Senescence was evaluated using the Beta-galactosidase assay (Senescence Cells Histochemical Staining Kit CS0030 (Sigma, St Louis, MO, USA) and clonogenic recovery assay as previously described.

#### 4.5.5. Cell Cycle Analysis

Cell cycle analysis was performed by flow cytometry of cells stained with Propidium Iodide (PI) (Thermo Fisher Scientific, Carlsbad, CA, USA) according to manufacturer’s instructions. Cells treated with the top PLK4i candidates and 0.1% DMSO (control) were fixed in 80% ethanol overnight, stained with PI, and then subjected to flow cytometric analysis using a BD Fortessa instrument (BD Biosciences, San Jose, CA, USA). Data was analyzed using Modfit LT from Verity Software House (Topsham, ME, USA). All experiments were performed in triplicate.

### 4.6. Characterization of KW-2449

#### 4.6.1. Kinase Activity Screening

The inhibitory activity of KW-2449 on various kinases was assessed through 486 biochemical kinase assays using 10 µM of the compound (SelectScreen Kinase Profiling Services—Thermo Fisher Scientific, Carlsbad, CA, USA). These assays utilized various formats appropriate to the kinase, its substrate, and its activity. The IC_50_ for selected kinases was further measured by 10-point titration.

#### 4.6.2. Drug Safety and Toxicology

Analysis of KW-2449 against a panel of P450s isoenzymes (1A2, 2B6, 2C8, 2C9, 2C19, 2D6, 2J2, 3A4, and 3A5) and substrates was performed using 10-point titrations, in duplicates to determine IC_50_ values (SelectScreen P450 Profiling Service—Thermo Fisher Scientific, Carlsbad, CA, USA). To determine if the compound binds the cardiac hERG channel, the IC_50_ value was quantitated using a 10-point titration, in duplicates in the Predictor hERG fluorescence polarization assay (SelectScreen hERG Screening Service—Thermo Fisher Scientific, Carlsbad, CA, USA).

## 5. Conclusions

Our analyses demonstrated that: (1) CFI-400437′s inhibition of PLK4 had the greatest impact overall, but similar to CFI-400945, its physiochemical properties are not optimal for brain exposure. Also, their phenotypic anti-cancer impact may, in part, be a consequence of the inhibition of AURKs, especially AURKB. (2) Centrinone and centrinone B are the most selective PLK4is but they are the least likely to penetrate the brain. (3) KW-2449, R1530, and axitinib are the ones predicted to have moderate-to-good BBB penetration. (4) The compound with the most chemically favorable molecular properties for brain exposure, KW-2449, was also the least selective for PLK4.

In conclusion, we showed that PLK4 can be inhibited by a variety of structurally disparate molecules, with varying degrees of in vitro phenotypic effects and that a selective PLK4i with favorable physiochemical properties for optimal brain exposure will be beneficial for the treatment of aggressive brain tumors with PLK4 overexpression. Finally, there is a need to improve the existing inhibitors for better selectivity and brain exposure and/or to develop new chemical entities with these desirable characteristics.

## 6. Patents

Simone Treiger Sredni and Tadanori Tomita, Northwestern University. INHIBITORS OF POLO-LIKE KINASE 4 (PLK4) FOR TREATING PEDIATRIC EMBRYONAL TUMORS. Patent application No. 20190070190. Filing Date: March 17, 2019.

## Figures and Tables

**Table 1:** *In silico* molecular docking scores of the protein kinase inhibitors to the binding cavity of PLK4 (GOLD 5.2, CCDC). Six simulations per kinase inhibitor were performed. Green—highest score; Red—lowest score.

	Best Score Found (per Simulation)						
	Docking 1	Docking 2	Docking 3	Docking 4	Docking 5	Docking 6	Mean
centrinone B	83.58	78.20	79.18	83.800	79.86	81.40	81.00
centrinone	80.10	85.33	73.78	79.86	81.74	70.87	78.61
alisertib	65.55	68.66	69.23	69.50	69.78	69.32	68.67
axitinib	64.46	63.49	64.41	65.92	62.97	65.55	64.47
CFI-400945	59.99	62.26	61.15	60.61	58.18	63.14	60.89
R-1530	55.23	62.02	62.05	62.01	62.03	62.01	60.89
CFI-400437	58.74	59.94	60.68	61.31	60.15	61.10	60.32
KW-2449	51.85	57.68	58.51	59.05	59.12	58.49	57.45

**Table 2:** (**A**) Physical multi-property analysis for each inhibitor, calculated using ACD Percepta 2016 (Build 2911). (**B**) LogBB values, obtained using ACD Labs Percepta software v16, for PKIs. Green—highest logBB; Red—lowest logBB.

A	CFI-400945			CFI-400437			Centrinone	
	CNS MPO Calculator			CNS MPO Calculator			CNS MPO Calculator	
	Property	Value		Property	Value		Property	Value
	clogP	5.05		clogP	4.07		clogP	4.03
	LogD_7.4_	4.98		LogD_7.4_	3.98		LogD_7.4_	4.03
	TPSA	79.48		TPSA	86.38		TPSA	204.84
	MW	534.65		MW	492.57		MW	633.65
	HBD	2		HBD	2		HBD	2
	pKa	6.6		pKa	7.2		pKa	2.3
					
	Centrinone B			R1530			KW-2449	
	CNS MPO Calculator			CNS MPO Calculator			CNS MPO Calculator	
	Property	Value		Property	Value		Property	Value
	clogP	4.31		clogP	3.82		clogP	2.56
	LogD_7.4_	4.31		LogD_7.4_	3.82		LogD_7.4_	2.14
	TPSA	195.61		TPSA	62.3		TPSA	61.02
	MW	631.68		MW	356.78		MW	332.40
	HBD	2		HBD	2		HBD	2
	pKa	4.2		pKa	5.6		pKa	8.3
						
	Axitinib			Alisertib		B	**Compound**	**logBB**
	CNS MPO Calculator			CNS MPO Calculator			CFI-400945	0.88
	Property	Value		Property	Value		R1530	0.3
	clogP	3.65		clogP	5.82		KW-2449	−0.07
	LogD_7.4_	3.65		LogD_7.4_	2.9		Centrinone B	−0.18
	TPSA	95.97		TPSA	105.93		Centrinone	−0.27
	MW	386.47		MW	518.92		Axitinib	−0.34
	HBD	2		HBD	2		CFI-400437	−0.73
	pKa	4.3		pKa	2.1		Alisertib	−0.96

**Table 3:** Half maximal inhibitory concentrations (IC_50_ nM) of each inhibitor on the activity of PLK4, AURKA, AURKB, and AURKC. Green—lowest IC_50_; Red—highest IC_50_.

	PLK4	AURKA	AURKB	AURKC
CFI-400437	1.55	37.2	13.1	4.88
centrinone	2.71	108	680	493
CFI-400945	4.85	188	70.7	106
axitinib	6.51	13.1	16.9	31.2
R1530	7.06	6.22	42.6	30.9
centrinone B	8.69	623	10,000	5810
KW-2449	52.6	45.8	23.8	23.2
alisertib	62.7	0.55	6.7	9.43

**Table 4:** Results from cell-based studies. In the first column: Half-maximal inhibitory concentrations (IC_50_) (SelectScreen, ThermoFisher, USA) from the kinase assay. MTT: IC_50_ of the proliferation assay; PB: IC_50_ of the PrestoBlue Viability assay (all values in nM) of each cell line (MON, BT-12, BT-16, DAOY, and D283). Green—lowest IC_50_; Red—highest IC_50_.

	SelectScreen IC_50_ (nM)	MON		BT-12		BT-16		DAOY		D283	
		MTT	PB	MTT	PB	MTT	PB	MTT	PB	MTT	PB
CFI-400437	1.55	722	640	61.6	604	1920	>10,000	684	768	>10,000	6304
centrinone	2.71	1840	5000	3875	3400	1557	2220	1250	4500	6950	6650
CFI-400945	4.85	67.4	5130	7250	8470	3300	5830	47.1	94	71	8400
axitinib	6.51	>10,000	4500	>10,000	>10,000	5950	>10,000	1147	1430	0.394	0.918
R-1530	7.06	3200	3750	>10,000	>10,000	3895	>10,000	829	1852	>10,000	>10,000
centrinone-B	8.69	>10,000	>10,000	5188	6320	5467	>10,000	>10,000	>10,000	1240	1740
KW-2449	52.6	2452	1743	>10,000	>10,000	>10,000	1840	>10,000	>10,000	8050	>10,000
alisertib	62.7	358	213	>10,000	>10,000	>10,000	>10,000	340	31	9533	9133

## Supplementary Materials

Supplementary materials can be found at https://www.mdpi.com/1422-0067/20/9/2112/s1.

## Abbreviations

AT/RT	Atypical Teratoid Rhabdoid Tumor
AURK	Aurora Kinase
AURKA	Aurora Kinase A
AURKB	Aurora Kinase B
AURKC	Aurora Kinase C
B3LYP	Becke-3-Lee Yang Parr
BBB	Blood Brain Barrier
CNS MPO	Central Nervous System Multi-Parameter Optimization
CYP450	Cytochrome P450
DFT	Density Functional Theory
EBT	Embryonal Brain Tumor
G3-MB	Group 3 MB
G4-MB	Group 4 MB
GBM	Glioblastoma multiforme
hERG	Human Ether-a-go-go-related gene
IC_50_	Half Maximal Inhibitory Concentration
LogP	Lipophilicity
MB	Medulloblastoma
MRT	Malignant Rhabdoid Tumor
MW	Molecular Weight
PB	Polo-box
PBD	Protein Data Bank
PI	Propidium Iodide
PK	Protein Kinase
PKI	Protein Kinase Inhibitor
PLK	Polo-like Kinase
PLK4	Polo-like kinase 4
PLK4i	Polo-like kinase 4 inhibitor
PSA	Polar Surface Area
RT	Rhabdoid Tumor
SAR	Structure-Activity Relationship
SHH	Sonic hedgehog
STM	Signal Transduction Modifiers
TPSA	Total Polar Surface Area
TRK	Tropomyosin Receptor Kinase

## References

[1] Bhullar K.S., Lagaron N.O., McGowan E.M., Parmar I., Jha A., Hubbard B.P., Rupasinghe H.P.V. (2018). Kinase-targeted cancer therapies: Progress, challenges and future directions. Mol. Cancer.

[2] Gross S., Rahal R., Stransky N., Lengauer C., Hoeflich K.P. (2015). Targeting cancer with kinase inhibitors. J. Clin. Investig..

[3] Nigg E.A., Holland A.J. (2018). Once and only once: Mechanisms of centriole duplication and their deregulation in disease. Nat. Rev. Mol. Cell Biol..

[4] Levine M.S., Bakker B., Boeckx B., Moyett J., Lu J., Vitre B., Spierings D.C., Lansdorp P.M., Cleveland D.W., Lambrechts D. (2017). Centrosome amplification is sufficient to promote spontaneous tumorigenesis in mammals. Dev. Cell.

[5] Chan J.Y. (2011). A clinical overview of centrosome amplification in human cancers. Int. J. Biol. Sci..

[6] Matthay K.K., Brisse H., Couanet D., Couturier J., Benard J., Mosseri V., Edeline V., Lumbroso J., Valteau-Couanet D., Michon J. (2003). Central nervous system metastases in neuroblastoma: Radiologic, clinical, and biologic features in 23 patients. Cancer.

[7] Fukasawa K. (2005). Centrosome amplification, chromosome instability and cancer development. Cancer Lett..

[8] Conduit P.T., Wainman A., Raff J.W. (2015). Centrosome function and assembly in animal cells. Nat. Rev. Mol. Cell Biol..

[9] Prakash A., Garcia-Moreno J.F., Brown J.A.L., Bourke E. (2018). Clinically applicable inhibitors impacting genome stability. Molecules.

[10] Godinho S.A., Pellman D. (2014). Causes and consequences of centrosome abnormalities in cancer. Philos. Trans. R. Soc. Lond. Ser. B. Biol. Sci..

[11] Johnson E.F., Stewart K.D., Woods K.W., Giranda V.L., Luo Y. (2007). Pharmacological and functional comparison of the polo-like kinase family: Insight into inhibitor and substrate specificity. Biochemistry.

[12] Slevin L.K., Nye J., Pinkerton D.C., Buster D.W., Rogers G.C., Slep K.C. (2012). The structure of the plk4 cryptic polo box reveals two tandem polo boxes required for centriole duplication. Structure (London, England: 1993).

[13] Klebba J.E., Buster D.W., McLamarrah T.A., Rusan N.M., Rogers G.C. (2015). Autoinhibition and relief mechanism for polo-like kinase 4. Proc. Natl. Acad. Sci. USA.

[14] Sillibourne J.E., Bornens M. (2010). Polo-like kinase 4: The odd one out of the family. Cell Division.

[15] Holland A.J., Cleveland D.W. (2014). Polo-like kinase 4 inhibition: A strategy for cancer therapy?. Cancer Cell.

[16] Holland A.J., Fachinetti D., Zhu Q., Bauer M., Verma I.M., Nigg E.A., Cleveland D.W. (2012). The autoregulated instability of polo-like kinase 4 limits centrosome duplication to once per cell cycle. Genes Dev..

[17] Guderian G., Westendorf J., Uldschmid A., Nigg E.A. (2010). Plk4 trans-autophosphorylation regulates centriole number by controlling betatrcp-mediated degradation. J. Cell Sci..

[18] Holland A.J., Lan W., Niessen S., Hoover H., Cleveland D.W. (2010). Polo-like kinase 4 kinase activity limits centrosome overduplication by autoregulating its own stability. J. Cell Biol..

[19] Sillibourne J.E., Tack F., Vloemans N., Boeckx A., Thambirajah S., Bonnet P., Ramaekers F.C., Bornens M., Grand-Perret T. (2010). Autophosphorylation of polo-like kinase 4 and its role in centriole duplication. Mol. Biol. Cell.

[20] Gonczy P. (2015). Centrosomes and cancer: Revisiting a long-standing relationship. Nat. Rev. Cancer.

[21] Bettencourt-Dias M., Hildebrandt F., Pellman D., Woods G., Godinho S.A. (2011). Centrosomes and cilia in human disease. Trends Genet..

[22] Dincer T., Yorgancioglu-Budak G., Olmez A., Er I., Dodurga Y., Ozdemir O.M., Toraman B., Yildirim A., Sabir N., Akarsu N.A. (2017). Analysis of centrosome and DNA damage response in plk4 associated seckel syndrome. Eur. J. Hum. Genet..

[23] Martin C.A., Ahmad I., Klingseisen A., Hussain M.S., Bicknell L.S., Leitch A., Nurnberg G., Toliat M.R., Murray J.E., Hunt D. (2014). Mutations in plk4, encoding a master regulator of centriole biogenesis, cause microcephaly, growth failure and retinopathy. Nat. Genet..

[24] Shaheen R., Al Tala S., Almoisheer A., Alkuraya F.S. (2014). Mutation in plk4, encoding a master regulator of centriole formation, defines a novel locus for primordial dwarfism. J. Med. Genet..

[25] Macmillan J.C., Hudson J.W., Bull S., Dennis J.W., Swallow C.J. (2001). Comparative expression of the mitotic regulators sak and plk in colorectal cancer. Ann. Surg. Oncol..

[26] Marina M., Saavedra H.I. (2014). Nek2 and plk4: Prognostic markers, drivers of breast tumorigenesis and drug resistance. Front. Biosci. (Landmark Ed.).

[27] Mason J.M., Lin D.C., Wei X., Che Y., Yao Y., Kiarash R., Cescon D.W., Fletcher G.C., Awrey D.E., Bray M.R. (2014). Functional characterization of cfi-400945, a polo-like kinase 4 inhibitor, as a potential anticancer agent. Cancer Cell.

[28] Kawakami M., Mustachio L.M., Zheng L., Chen Y., Rodriguez-Canales J., Mino B., Kurie J.M., Roszik J., Villalobos P.A., Thu K.L. (2018). Polo-like kinase 4 inhibition produces polyploidy and apoptotic death of lung cancers. Proc. Natl. Acad. Sci. USA.

[29] Denu R.A., Shabbir M., Nihal M., Singh C.K., Longley B.J., Burkard M.E., Ahmad N. (2018). Centriole overduplication is the predominant mechanism leading to centrosome amplification in melanoma. Mol. Cancer Res..

[30] Hudnall S.D., Meng H., Lozovatsky L., Li P., Strout M., Kleinstein S.H. (2016). Recurrent genetic defects in classical hodgkin lymphoma cell lines. Leuk. Lymphoma.

[31] Roberto G.M., Engel E.E., Scrideli C.A., Tone L.G., Brassesco M.S. (2018). Downregulation of mir-10b* is correlated with altered expression of mitotic kinases in osteosarcoma. Pathol. Res. Pract..

[32] Shinmura K., Kurabe N., Goto M., Yamada H., Natsume H., Konno H., Sugimura H. (2014). Plk4 overexpression and its effect on centrosome regulation and chromosome stability in human gastric cancer. Mol. Biol. Rep..

[33] Lohse I., Mason J., Cao P.M., Pintilie M., Bray M., Hedley D.W. (2017). Activity of the novel polo-like kinase 4 inhibitor cfi-400945 in pancreatic cancer patient-derived xenografts. Oncotarget.

[34] Zhang Z., Wang Z., Huang K., Liu Y., Wei C., Zhou J., Zhang W., Wang Q., Liang H., Zhang A. (2019). Plk4 is a determinant of temozolomide sensitivity through phosphorylation of ikbke in glioblastoma. Cancer Lett..

[35] Liu L., Zhang C.Z., Cai M., Fu J., Chen G.G., Yun J. (2012). Downregulation of polo-like kinase 4 in hepatocellular carcinoma associates with poor prognosis. PLoS ONE.

[36] Sredni S.T., Bailey A.W., Suri A., Hashizume R., He X., Louis N., Gokirmak T., Piper D.R., Watterson D.M., Tomita T. (2017). Inhibition of polo-like kinase 4 (plk4): A new therapeutic option for rhabdoid tumors and pediatric medulloblastoma. Oncotarget.

[37] Sredni S.T., Suzuki M., Yang J.P., Topczewski J., Bailey A.W., Gokirmak T., Gross J.N., de Andrade A., Kondo A., Piper D.R. (2017). A functional screening of the kinome identifies the polo-like kinase 4 as a potential therapeutic target for malignant rhabdoid tumors, and possibly, other embryonal tumors of the brain. Pediatric Blood Cancer.

[38] Sredni S.T., Tomita T. (2017). The polo-like kinase 4 gene (plk4) is overexpressed in pediatric medulloblastoma. Child’s Nerv. Syst. ChNS Off. J. Int. Soc. Pediatric Neurosurg..

[39] Bailey A.W., Suri A., Chou P.M., Pundy T., Gadd S., Raimondi S.L., Tomita T., Sredni S.T. (2018). Polo-like kinase 4 (plk4) is overexpressed in central nervous system neuroblastoma (cns-nb). Bioengineering.

[40] Nemes K., Fruhwald M.C. (2018). Emerging therapeutic targets for the treatment of malignant rhabdoid tumors. Expert Opin. Ther. Targets.

[41] Sredni S.T., Tomita T. (2015). Rhabdoid tumor predisposition syndrome. Pediatric Dev. Pathol. Off. J. Soc. Pediatric Pathol. Paediatr. Pathol. Soc..

[42] Fruhwald M.C., Biegel J.A., Bourdeaut F., Roberts C.W., Chi S.N. (2016). Atypical teratoid/rhabdoid tumors-current concepts, advances in biology, and potential future therapies. Neuro-Oncology.

[43] Grupenmacher A.T., Halpern A.L., Bonaldo Mde F., Huang C.C., Hamm C.A., de Andrade A., Tomita T., Sredni S.T. (2013). Study of the gene expression and microrna expression profiles of malignant rhabdoid tumors originated in the brain (at/rt) and in the kidney (rtk). Child’s Nerv. Syst. ChNS Off. J. Int. Soc. Pediatric Neurosurg..

[44] Ostrom Q.T., de Blank P.M., Kruchko C., Petersen C.M., Liao P., Finlay J.L., Stearns D.S., Wolff J.E., Wolinsky Y., Letterio J.J. (2015). Alex’s lemonade stand foundation infant and childhood primary brain and central nervous system tumors diagnosed in the united states in 2007-2011. Neuro-Oncology.

[45] Torchia J., Picard D., Lafay-Cousin L., Hawkins C.E., Kim S.K., Letourneau L., Ra Y.S., Ho K.C., Chan T.S., Sin-Chan P. (2015). Molecular subgroups of atypical teratoid rhabdoid tumours in children: An integrated genomic and clinicopathological analysis. Lancet. Oncol..

[46] Torchia J., Golbourn B., Feng S., Ho K.C., Sin-Chan P., Vasiljevic A., Norman J.D., Guilhamon P., Garzia L., Agamez N.R. (2016). Integrated (epi)-genomic analyses identify subgroup-specific therapeutic targets in cns rhabdoid tumors. Cancer Cell.

[47] Johann P.D., Erkek S., Zapatka M., Kerl K., Buchhalter I., Hovestadt V., Jones D.T.W., Sturm D., Hermann C., Segura Wang M. (2016). Atypical teratoid/rhabdoid tumors are comprised of three epigenetic subgroups with distinct enhancer landscapes. Cancer Cell.

[48] Batora N.V., Sturm D., Jones D.T., Kool M., Pfister S.M., Northcott P.A. (2014). Transitioning from genotypes to epigenotypes: Why the time has come for medulloblastoma epigenomics. Neuroscience.

[49] Dubuc A.M., Remke M., Korshunov A., Northcott P.A., Zhan S.H., Mendez-Lago M., Kool M., Jones D.T., Unterberger A., Morrissy A.S. (2013). Aberrant patterns of h3k4 and h3k27 histone lysine methylation occur across subgroups in medulloblastoma. Acta Neuropathol..

[50] Jones D.T., Northcott P.A., Kool M., Pfister S.M. (2013). The role of chromatin remodeling in medulloblastoma. Brain Pathol. (Zurich, Switzerland).

[51] Remke M., Ramaswamy V., Taylor M.D. (2013). Medulloblastoma molecular dissection: The way toward targeted therapy. Curr. Opin. Oncol..

[52] Parsons D.W., Li M., Zhang X., Jones S., Leary R.J., Lin J.C., Boca S.M., Carter H., Samayoa J., Bettegowda C. (2011). The genetic landscape of the childhood cancer medulloblastoma. Science.

[53] Huether R., Dong L., Chen X., Wu G., Parker M., Wei L., Ma J., Edmonson M.N., Hedlund E.K., Rusch M.C. (2014). The landscape of somatic mutations in epigenetic regulators across 1000 paediatric cancer genomes. Nat. Commun..

[54] Ramaswamy V., Taylor M.D. (2017). Medulloblastoma: From myth to molecular. J. Clin. Oncol. Off. J. Am. Soc. Clin. Oncol..

[55] Sampson P.B., Liu Y., Forrest B., Cumming G., Li S.W., Patel N.K., Edwards L., Laufer R., Feher M., Ban F. (2015). The discovery of polo-like kinase 4 inhibitors: Identification of (1r,2s).2-(3-((e).4-(((cis).2,6-dimethylmorpholino)methyl)styryl). 1h.Indazol-6-yl)-5′-methoxyspiro[cyclopropane-1,3′-indolin]-2′-one (cfi-400945) as a potent, orally active antitumor agent. J. Med. Chem..

[56] Sampson P.B., Liu Y., Patel N.K., Feher M., Forrest B., Li S.W., Edwards L., Laufer R., Lang Y., Ban F. (2015). The discovery of polo-like kinase 4 inhibitors: Design and optimization of spiro[cyclopropane-1,3′[3h]indol]-2′(1′h).Ones as orally bioavailable antitumor agents. J. Med. Chem..

[57] Liu J.J., Higgins B., Ju G., Kolinsky K., Luk K.C., Packman K., Pizzolato G., Ren Y., Thakkar K., Tovar C. (2013). Discovery of a highly potent, orally active mitosis/angiogenesis inhibitor r1530 for the treatment of solid tumors. ACS Med. Chem. Lett..

[58] Peart P.A., Tovar J.D. (2010). Poly(cyclopropenone)s: Formal inclusion of the smallest huckel aromatic into pi-conjugated polymers. J. Org. Chem..

[59] Wong Y.L., Anzola J.V., Davis R.L., Yoon M., Motamedi A., Kroll A., Seo C.P., Hsia J.E., Kim S.K., Mitchell J.W. (2015). Cell biology. Reversible centriole depletion with an inhibitor of polo-like kinase 4. Science.

[60] Laufer R., Forrest B., Li S.W., Liu Y., Sampson P., Edwards L., Lang Y., Awrey D.E., Mao G., Plotnikova O. (2013). The discovery of plk4 inhibitors: (e)-3-((1h-indazol-6-yl)methylene)indolin-2-ones as novel antiproliferative agents. J. Med. Chem..

[61] Wilmes L.J., Pallavicini M.G., Fleming L.M., Gibbs J., Wang D., Li K.L., Partridge S.C., Henry R.G., Shalinsky D.R., Hu-Lowe D. (2007). Ag-013736, a novel inhibitor of vegf receptor tyrosine kinases, inhibits breast cancer growth and decreases vascular permeability as detected by dynamic contrast-enhanced magnetic resonance imaging. Magn. Reson. Imaging.

[62] Hu-Lowe D.D., Zou H.Y., Grazzini M.L., Hallin M.E., Wickman G.R., Amundson K., Chen J.H., Rewolinski D.A., Yamazaki S., Wu E.Y. (2008). Nonclinical antiangiogenesis and antitumor activities of axitinib (ag-013736), an oral, potent, and selective inhibitor of vascular endothelial growth factor receptor tyrosine kinases 1, 2, 3. Clin. Cancer Res..

[63] Pratz K.W., Cortes J., Roboz G.J., Rao N., Arowojolu O., Stine A., Shiotsu Y., Shudo A., Akinaga S., Small D. (2009). A pharmacodynamic study of the flt3 inhibitor kw-2449 yields insight into the basis for clinical response. Blood.

[64] Shiotsu Y., Kiyoi H., Ishikawa Y., Tanizaki R., Shimizu M., Umehara H., Ishii K., Mori Y., Ozeki K., Minami Y. (2009). Kw-2449, a novel multikinase inhibitor, suppresses the growth of leukemia cells with flt3 mutations or t315i-mutated bcr/abl translocation. Blood.

[65] Wetmore C., Boyett J., Li S., Lin T., Bendel A., Gajjar A., Orr B.A. (2015). Alisertib is active as single agent in recurrent atypical teratoid rhabdoid tumors in 4 children. Neuro-Oncology.

[66] Melichar B., Adenis A., Lockhart A.C., Bennouna J., Dees E.C., Kayaleh O., Obermannova R., DeMichele A., Zatloukal P., Zhang B. (2015). Safety and activity of alisertib, an investigational aurora kinase a inhibitor, in patients with breast cancer, small-cell lung cancer, non-small-cell lung cancer, head and neck squamous-cell carcinoma, and gastro-oesophageal adenocarcinoma: A five-arm phase 2 study. Lancet. Oncol..

[67] Liu Z., Lei Q., Wei W., Xiong L., Shi Y., Yan G., Gao C., Ye T., Wang N., Yu L. (2017). Synthesis and biological evaluation of (e)-4-(3-arylvinyl-1h-indazol-6-yl)pyrimidin-2-amine derivatives as plk4 inhibitors for the treatment of breast cancer. RSC Adv..

[68] Escudier B., Gore M. (2011). Axitinib for the management of metastatic renal cell carcinoma. Drugs R&D.

[69] Gross-Goupil M., Francois L., Quivy A., Ravaud A. (2013). Axitinib: A review of its safety and efficacy in the treatment of adults with advanced renal cell carcinoma. Clin. Med. Insights. Oncol..

[70] Cohen E.E.W., Rosen L.S., Vokes E.E., Kies M.S., Forastiere A.A., Worden F.P., Kane M.A., Sherman E., Kim S., Bycott P. (2008). Axitinib is an active treatment for all histologic subtypes of advanced thyroid cancer: Results from a phase ii study. J. Clin. Oncol. Off. J. Am. Soc. Clin. Oncol..

[71] Schiller J.H., Larson T., Ou S.I., Limentani S.A., Sandler A.B., Vokes E.E., Kim S., Liau K.F., Bycott P.W., Olszanski A.J. (2007). Efficacy and safety of axitinib (ag-013736; ag) in patients (pts) with advanced non-small cell lung cancer (nsclc): A phase ii trial. J. Clin. Oncol..

[72] Fruehauf J.P., Lutzky J., McDermott D.F., Brown C.K., Pithavala Y.K., Bycott P.W., Shalinsky D., Liau K.F., Niethammer A., Rixe O. (2008). Axitinib (ag-013736) in patients with metastatic melanoma: A phase ii study. J. Clin. Oncol..

[73] Sells T.B., Chau R., Ecsedy J.A., Gershman R.E., Hoar K., Huck J., Janowick D.A., Kadambi V.J., LeRoy P.J., Stirling M. (2015). Mln8054 and alisertib (mln8237): Discovery of selective oral aurora a inhibitors. ACS Med. Chem. Lett..

[74] Venkataraman S., Alimova I., Tello T., Harris P.S., Knipstein J.A., Donson A.M., Foreman N.K., Liu A.K., Vibhakar R. (2012). Targeting aurora kinase a enhances radiation sensitivity of atypical teratoid rhabdoid tumor cells. J. Neuro-Oncology.

[75] Pardridge W.M. (2005). The blood-brain barrier: Bottleneck in brain drug development. NeuroRx J. Am. Soc. Exp. Neurother..

[76] Gunosewoyo H., Yu L., Munoz L., Kassiou M. (2017). Kinase targets in cns drug discovery. Future Med. Chem..

[77] Heffron T.P. (2016). Small molecule kinase inhibitors for the treatment of brain cancer. J. Med. Chem..

[78] Chico L.K., Van Eldik L.J., Watterson D.M. (2009). Targeting protein kinases in central nervous system disorders. Nat. Rev. Drug Discov..

[79] Wager T.T., Hou X., Verhoest P.R., Villalobos A. (2016). Central nervous system multiparameter optimization desirability: Application in drug discovery. ACS Chem. Neurosci..

[80] Wager T.T., Hou X., Verhoest P.R., Villalobos A. (2010). Moving beyond rules: The development of a central nervous system multiparameter optimization (cns mpo) approach to enable alignment of druglike properties. ACS Chem. Neurosci..

[81] Levinson N.M. (2018). The multifaceted allosteric regulation of aurora kinase a. Biochem. J..

[82] Brown J.R., Koretke K.K., Birkeland M.L., Sanseau P., Patrick D.R. (2004). Evolutionary relationships of aurora kinases: Implications for model organism studies and the development of anti-cancer drugs. BMC Evol. Biol..

[83] Lordier L., Chang Y., Jalil A., Aurade F., Garcon L., Lecluse Y., Larbret F., Kawashima T., Kitamura T., Larghero J. (2010). Aurora b is dispensable for megakaryocyte polyploidization, but contributes to the endomitotic process. Blood.

[84] Noronha S., Alt L.A.C., Scimeca T.E., Zarou O., Obrzut J., Zanotti B., Hayward E.A., Pillai A., Mathur S., Rojas J. (2018). Preclinical evaluation of the aurora kinase inhibitors amg 900, azd1152-hqpa, and mk-5108 on sw-872 and 93t449 human liposarcoma cells. In Vitro Cell. Dev. Biol. Anim..

[85] Ducat D., Zheng Y. (2004). Aurora kinases in spindle assembly and chromosome segregation. Exp. Cell Res..

[86] Hauf S., Cole R.W., LaTerra S., Zimmer C., Schnapp G., Walter R., Heckel A., van Meel J., Rieder C.L., Peters J.M. (2003). The small molecule hesperadin reveals a role for aurora b in correcting kinetochore-microtubule attachment and in maintaining the spindle assembly checkpoint. J. Cell Biol..

[87] Mitcheson J.S., Chen J., Lin M., Culberson C., Sanguinetti M.C. (2000). A structural basis for drug-induced long qt syndrome. Proc. Natl. Acad. Sci. USA.

[88] Lynch T., Price A. (2007). The effect of cytochrome P450 metabolism on drug response, interactions, and adverse effects. Am. Fam. Physician..

[89] Kemper E.M., Leenders W., Kusters B., Lyons S., Buckle T., Heerschap A., Boogerd W., Beijnen J.H., van Tellingen O. (2006). Development of luciferase tagged brain tumour models in mice for chemotherapy intervention studies. Eur. J. Cancer (Oxford, England: 1990).

[90] Bovée J.V.M.G., van Royen M., Bardoel A.F.J., Rosenberg C., Cornelisse C.J., Cleton-Jansen A.-M., Hogendoorn P.C.W. (2000). Near-haploidy and subsequent polyploidization characterize the progression of peripheral chondrosarcoma. Am. J. Pathol..

[91] Duensing A., Duensing S. (2010). Centrosomes, polyploidy and cancer. Adv. Exp. Med. Biol..

[92] Mosieniak G., Sikora E. (2010). Polyploidy: The link between senescence and cancer. Curr. Pharm. Des..

[93] Davoli T., de Lange T. (2011). The causes and consequences of polyploidy in normal development and cancer. Annu. Rev. Cell Dev. Biol..

[94] Hau P.M., Siu W.Y., Wong N., Lai P.B., Poon R.Y. (2006). Polyploidization increases the sensitivity to DNA-damaging agents in mammalian cells. FEBS Lett..

[95] Yan M., Wang C., He B., Yang M., Tong M., Long Z., Liu B., Peng F., Xu L., Zhang Y. (2016). Aurora-a kinase: A potent oncogene and target for cancer therapy. Med. Res. Rev..

[96] Zou X., Qu M., Fang F., Fan Z., Chen L., Yue W., Xie X., Pei X. (2017). Small molecule supplements improve cultured megakaryocyte polyploidization by modulating multiple cell cycle regulators. BioMed Res. Int..

[97] Harrington E.A., Bebbington D., Moore J., Rasmussen R.K., Ajose-Adeogun A.O., Nakayama T., Graham J.A., Demur C., Hercend T., Diu-Hercend A. (2004). Vx-680, a potent and selective small-molecule inhibitor of the aurora kinases, suppresses tumor growth in vivo. Nat. Med..

[98] Versteege I., Medjkane S., Rouillard D., Delattre O. (2002). A key role of the hsnf5/ini1 tumour suppressor in the control of the g1-s transition of the cell cycle. Oncogene.

[99] Singh A., Lun X., Jayanthan A., Obaid H., Ruan Y., Strother D., Chi S.N., Smith A., Forsyth P., Narendran A. (2013). Profiling pathway-specific novel therapeutics in preclinical assessment for central nervous system atypical teratoid rhabdoid tumors (cns atrt): Favorable activity of targeting egfr- erbb2 signaling with lapatinib. Mol. Oncol..

