# Supplementary material for: Evaluation of Protein Kinase Inhibitors with PLK4 Cross-Over Potential in a Pre-Clinical Model of Cancer
Source: Int J Mol Sci. 2019 Apr 29;20(9):2112. doi: 10.3390/ijms20092112 (PMC6540285; doi:10.3390/ijms20092112)
Supplement: Supplementary file 1 [file ijms-20-02112-s001.zip › Supplementary Figure and Table/Supplementary Table 1.pdf]

Kinase	Technology	% Inhibition Avg
AAK1	LanthaScreen	87
ABL1	ZLYTE	100
ABL1 E255K	ZLYTE	99
ABL1 F317I	ZLYTE	94
ABL1 F317L	ZLYTE	98
ABL1 G250E	ZLYTE	100
ABL1 H396P	LanthaScreen	78
ABL1 M351T	LanthaScreen	52
ABL1 Q252H	LanthaScreen	85
ABL1 T315I	ZLYTE	101
ABL1 Y253F	ZLYTE	100
ABL2 (Arg)	ZLYTE	98
ACVR1 (ALK2)	LanthaScreen	94
ACVR1 (ALK2) R206H	LanthaScreen	69
ACVR1B (ALK4)	ZLYTE	39
ACVR2A	LanthaScreen	86
ACVR2B	LanthaScreen	94
ACVRL1 (ALK1)	LanthaScreen	86
ADCK3	LanthaScreen	-2
ADRBK1 (GRK2)	ZLYTE	38
ADRBK2 (GRK3)	ZLYTE	31
AKT1 (PKB alpha)	ZLYTE	24
AKT2 (PKB beta)	ZLYTE	29
AKT3 (PKB gamma)	ZLYTE	11
ALK	ZLYTE	101
ALK C1156Y	LanthaScreen	76
ALK F1174L	LanthaScreen	56
ALK L1196M	LanthaScreen	13
ALK R1275Q	LanthaScreen	52
ALK T1151_L1152insT	LanthaScreen	26
AMPK (A1/B1/G2)	LanthaScreen	85
AMPK (A1/B1/G3)	LanthaScreen	71
AMPK (A1/B2/G1)	LanthaScreen	77
AMPK (A1/B2/G2)	ZLYTE	99
AMPK (A1/B2/G3)	ZLYTE	99
AMPK (A2/B1/G2)	ZLYTE	97
AMPK (A2/B1/G3)	ZLYTE	96
AMPK (A2/B2/G1)	LanthaScreen	75
AMPK (A2/B2/G2)	LanthaScreen	78
AMPK (A2/B2/G3)	ZLYTE	98
AMPK A1/B1/G1	ZLYTE	97
AMPK A2/B1/G1	ZLYTE	99
AURKA (Aurora A)	ZLYTE	98
AURKB (Aurora B)	ZLYTE	102
AURKC (Aurora C)	ZLYTE	98
AXL	ZLYTE	95
AXL R499C	LanthaScreen	45
BLK	ZLYTE	89
BMPR1A (ALK3)	LanthaScreen	98
BMPR1B (ALK6)	LanthaScreen	90
BMPR2	LanthaScreen	113
BMX	ZLYTE	88
BRAF	LanthaScreen	-1
BRAF	ZLYTE	67
BRAF V599E	LanthaScreen	6
BRAF V599E	ZLYTE	60
BRSK1 (SAD1)	ZLYTE	99
BRSK2	LanthaScreen	7
BTK	ZLYTE	99
CAMK1 (CaMK1)	Adapta	89
CAMK1D (CaMKI delta)	ZLYTE	80
CAMK1G (CAMKI gamma)	ZLYTE	71
CAMK2A (CaMKII alpha)	ZLYTE	67
CAMK2B (CaMKII beta)	ZLYTE	32
CAMK2D (CaMKII delta)	ZLYTE	96
CAMK2G (CaMKII gamma)	LanthaScreen	-110
CAMK4 (CaMKIV)	ZLYTE	52
CAMKK1 (CAMKKA)	LanthaScreen	69
CAMKK2 (CaMKK beta)	LanthaScreen	86
CASK	LanthaScreen	-63
CDC42 BPA (MRCKA)	ZLYTE	32
CDC42 BPB (MRCKB)	ZLYTE	27
CDC42 BPG (MRCKG)	ZLYTE	68
CDC7/DBF4	LanthaScreen	16
CDK1/cyclin B	ZLYTE	88
CDK11 (Inactive)	LanthaScreen	29
CDK11/cyclin C	LanthaScreen	53
CDK13/cyclin K	LanthaScreen	70
CDK14 (PFTK1)/cyclin Y	LanthaScreen	66
CDK16 (PCTK1)/cyclin Y	LanthaScreen	81
CDK17/cyclin Y	ZLYTE	97
CDK18/cyclin Y	ZLYTE	88
CDK2/cyclin A	ZLYTE	95
CDK2/cyclin A1	LanthaScreen	50
CDK2/cyclin E1	LanthaScreen	36
CDK2/cyclin O	LanthaScreen	-32
CDK3/cyclin E1	LanthaScreen	48
CDK4/cyclin D1	Adapta	85
CDK4/cyclin D3	Adapta	83
CDK5 (Inactive)	LanthaScreen	8
CDK5/p25	ZLYTE	97
CDK5/p35	ZLYTE	94
CDK6/cyclin D1	Adapta	82
CDK7/cyclin H/MNAT1	Adapta	98
CDK8/cyclin C	LanthaScreen	38
CDK9 (Inactive)	LanthaScreen	2
CDK9/cyclin K	LanthaScreen	38
CDK9/cyclin T1	Adapta	82
CDKL5	ZLYTE	56
CHEK1 (CHK1)	ZLYTE	99
CHEK2 (CHK2)	ZLYTE	100
CHUK (IKK alpha)	Adapta	96
CLK1	ZLYTE	99
CLK2	ZLYTE	97
CLK3	ZLYTE	66
CLK4	LanthaScreen	84
CSF1R (FMS)	ZLYTE	99
CSK	ZLYTE	71
CSNK1A1 (CK1 alpha 1)	ZLYTE	33
CSNK1A1L	ZLYTE	20
CSNK1D (CK1 delta)	ZLYTE	53
CSNK1E (CK1 epsilon)	ZLYTE	67
CSNK1E (CK1 epsilon) R178C	ZLYTE	71
CSNK1G1 (CK1 gamma 1)	ZLYTE	74
CSNK1G2 (CK1 gamma 2)	ZLYTE	87
CSNK1G3 (CK1 gamma 3)	ZLYTE	77
CSNK2A1 (CK2 alpha 1)	ZLYTE	18
CSNK2A2 (CK2 alpha 2)	ZLYTE	38
DAPK1	Adapta	90
DAPK2	LanthaScreen	64
DAPK3 (ZIPK)	ZLYTE	67
DCAMKL1 (DCLK1)	ZLYTE	57
DCAMKL2 (DCK2)	ZLYTE	56
DDR1	LanthaScreen	55
DDR2	LanthaScreen	53
DDR2 N456S	LanthaScreen	-96
DDR2 T654M	LanthaScreen	84
DMPK	LanthaScreen	77
DNA-PK	ZLYTE	24
DYRK1A	ZLYTE	90
DYRK1B	ZLYTE	91
DYRK2	LanthaScreen	47
DYRK3	ZLYTE	86
DYRK4	ZLYTE	16
EEF2K	ZLYTE	-1
EGFR (ErbB1)	ZLYTE	49
EGFR (ErbB1) C797S	ZLYTE	51
EGFR (ErbB1) d746-750	LanthaScreen	-17
EGFR (ErbB1) d747-749 A750P	LanthaScreen	30
EGFR (ErbB1) G719C	ZLYTE	43
EGFR (ErbB1) G719S	ZLYTE	68
EGFR (ErbB1) L858R	ZLYTE	48
EGFR (ErbB1) L861Q	ZLYTE	52
EGFR (ErbB1) T790M	ZLYTE	96
EGFR (ErbB1) T790M C797S L858R	ZLYTE	96
EGFR (ErbB1) T790M L858R	ZLYTE	97
EIF2AK2 (PKR)	LanthaScreen	7
EPHA1	ZLYTE	99
EPHA2	ZLYTE	98

Kinase	Technology	% Inhibition Avg
EPHA3	LanthaScreen	61
EPHA4	ZLYTE	83
EPHA5	ZLYTE	84
EPHA6	LanthaScreen	73
EPHA7	LanthaScreen	66
EPHA8	ZLYTE	79
EPHB1	ZLYTE	97
EPHB2	ZLYTE	95
EPHB3	ZLYTE	21
EPHB4	ZLYTE	87
ERBB2 (HER2)	ZLYTE	4
ERBB4 (HER4)	ZLYTE	55
ERN1	LanthaScreen	32
ERN2	LanthaScreen	74
FER	ZLYTE	98
FES (FPS)	ZLYTE	98
FGFR1	ZLYTE	96
FGFR1 V561M	LanthaScreen	30
FGFR2	ZLYTE	97
FGFR2 N549H	ZLYTE	94
FGFR3	ZLYTE	97
FGFR3 G697C	LanthaScreen	78
FGFR3 K650E	ZLYTE	97
FGFR3 K650M	LanthaScreen	22
FGFR3 V555M	ZLYTE	100
FGFR4	ZLYTE	70
FGR	ZLYTE	99
FLT1 (VEGFR1)	ZLYTE	87
FLT3	ZLYTE	100
FLT3 D835Y	ZLYTE	100
FLT3 ITD	LanthaScreen	5
FLT4 (VEGFR3)	ZLYTE	95
FRAP1 (mTOR)	ZLYTE	9
FRK (PTK5)	ZLYTE	90
FYN	ZLYTE	94
FYN A	LanthaScreen	72
GAK	LanthaScreen	67
GRK1	LanthaScreen	25
GRK4	ZLYTE	61
GRK5	ZLYTE	17
GRK6	ZLYTE	62
GRK7	ZLYTE	41
GSG2 (Haspin)	Adapta	74
GSK3A (GSK3 alpha)	ZLYTE	63
GSK3B (GSK3 beta)	ZLYTE	66
HCK	ZLYTE	90
HIPK1 (Myak)	ZLYTE	70
HIPK2	ZLYTE	92
HIPK3 (YAK1)	ZLYTE	53
HIPK4	ZLYTE	85
HUNK	LanthaScreen	84
ICK	LanthaScreen	74
IGF1R	ZLYTE	95
IKBKB (IKK beta)	ZLYTE	66
IKBKE (IKK epsilon)	ZLYTE	92
INSR	ZLYTE	96
INSRR (IRR)	ZLYTE	96
IRAK1	Adapta	99
IRAK3	LanthaScreen	31
IRAK4	ZLYTE	77
ITK	ZLYTE	99
JAK1	ZLYTE	94
JAK2	ZLYTE	98
JAK2 JH1 JH2	ZLYTE	96
JAK2 JH1 JH2 V617F	ZLYTE	94
JAK3	ZLYTE	98
KDR (VEGFR2)	ZLYTE	99
KIT	ZLYTE	91
KIT A829P	LanthaScreen	67
KIT D816H	LanthaScreen	42
KIT D816V	LanthaScreen	40
KIT D820E	LanthaScreen	81
KIT N822K	LanthaScreen	68
KIT T670E	LanthaScreen	89

Kinase	Technology	% Inhibition Avg
KIT V559D	ZLYTE	91
KIT V559D T670I	LanthaScreen	67
KIT V559D V654A	ZLYTE	77
KIT V560G	ZLYTE	84
KIT V654A	LanthaScreen	68
KIT Y823D	LanthaScreen	61
KSR2	ZLYTE	5
LATS1	LanthaScreen	-17
LATS2	LanthaScreen	68
LCK	ZLYTE	97
LIMK1	LanthaScreen	32
LIMK2	LanthaScreen	24
LRRK2	Adapta	100
LRRK2 FL	Adapta	100
LRRK2 G2019S	Adapta	100
LRRK2 G2019S FL	Adapta	100
LRRK2 I2020T	Adapta	100
LRRK2 R1441C	Adapta	100
LTK (TYK1)	ZLYTE	90
LYN A	ZLYTE	89
LYN B	ZLYTE	94
MAP2K1 (MEK1)	LanthaScreen	54
MAP2K1 (MEK1)	ZLYTE	72
MAP2K1 (MEK1) S218D S222D	LanthaScreen	66
MAP2K2 (MEK2)	LanthaScreen	73
MAP2K2 (MEK2)	ZLYTE	75
MAP2K4 (MEK4)	LanthaScreen	79
MAP2K5 (MEK5)	LanthaScreen	84
MAP2K6 (MKK6)	LanthaScreen	56
MAP2K6 (MKK6)	ZLYTE	79
MAP2K6 (MKK6) S207E T211E	LanthaScreen	82
MAP3K10 (MLK2)	LanthaScreen	87
MAP3K11 (MLK3)	LanthaScreen	86
MAP3K14 (NIK)	LanthaScreen	90
MAP3K19 (YSK4)	ZLYTE	98
MAP3K2 (MEKK2)	LanthaScreen	86
MAP3K3 (MEKK3)	LanthaScreen	82
MAP3K5 (ASK1)	LanthaScreen	63
MAP3K7/MAP3K7IP1 (TAK1-TAB1)	LanthaScreen	26
MAP3K8 (COT)	ZLYTE	54
MAP3K9 (MLK1)	ZLYTE	102
MAP4K1 (HPK1)	LanthaScreen	76
MAP4K2 (GCK)	ZLYTE	101
MAP4K3 (GLK)	LanthaScreen	72
MAP4K4 (HGK)	ZLYTE	98
MAP4K5 (KHS1)	ZLYTE	102
MAPK1 (ERK2)	ZLYTE	45
MAPK10 (JNK3)	LanthaScreen	88
MAPK10 (JNK3)	ZLYTE	59
MAPK11 (p38 beta)	ZLYTE	90
MAPK12 (p38 gamma)	ZLYTE	23
MAPK13 (p38 delta)	ZLYTE	30
MAPK14 (p38 alpha)	ZLYTE	33
MAPK14 (p38 alpha) Direct	ZLYTE	44
MAPK15 (ERK7)	LanthaScreen	-49
MAPK3 (ERK1)	ZLYTE	41
MAPK7 (ERK5)	ZLYTE	6
MAPK8 (JNK1)	LanthaScreen	81
MAPK8 (JNK1)	ZLYTE	96
MAPK9 (JNK2)	LanthaScreen	79
MAPK9 (JNK2)	ZLYTE	87
MAPKAPK2	ZLYTE	28
MAPKAPK3	ZLYTE	4
MAPKAPK5 (PRAK)	ZLYTE	87
MARK1 (MARK)	ZLYTE	99
MARK2	ZLYTE	98
MARK3	ZLYTE	99
MARK4	ZLYTE	101
MASTL	LanthaScreen	83
MATK (HYL)	ZLYTE	37
MELK	ZLYTE	101
MERTK (cMER)	ZLYTE	93
MERTK (cMER) A708S	LanthaScreen	74
MET (cMet)	ZLYTE	95

Kinase	Technology	% Inhibition Avg
MET (cMet) Y1235D	ZLYTE	88
MET D1228H	LanthaScreen	75
MET M1250T	ZLYTE	96
MINK1	ZLYTE	102
MKNK1 (MNK1)	ZLYTE	52
MKNK2 (MNK2)	LanthaScreen	68
MLCK (MLCK2)	LanthaScreen	77
MLK4	LanthaScreen	17
MST1R (RON)	ZLYTE	97
MST4	ZLYTE	92
MUSK	ZLYTE	90
MYLK (MLCK)	LanthaScreen	80
MYLK2 (skMLCK)	ZLYTE	100
MYLK4	LanthaScreen	71
MYO3A (MYO3 alpha)	LanthaScreen	59
MYO3B (MYO3 beta)	LanthaScreen	88
NEK1	ZLYTE	67
NEK2	ZLYTE	47
NEK4	ZLYTE	96
NEK6	ZLYTE	10
NEK7	ZLYTE	7
NEK8	LanthaScreen	30
NEK9	ZLYTE	70
NIM1K	ZLYTE	79
NLK	LanthaScreen	-2
NTRK1 (TRKA)	ZLYTE	98
NTRK2 (TRKB)	ZLYTE	99
NTRK3 (TRKC)	ZLYTE	100
NUAK1 (ARK5)	Adapta	100
NUAK2	LanthaScreen	62
PAK1	ZLYTE	72
PAK2 (PAK65)	ZLYTE	57
PAK3	ZLYTE	52
PAK4	ZLYTE	94
PAK6	ZLYTE	60
PAK7 (KIAA1264)	ZLYTE	98
PASK	ZLYTE	10
PDGFRA (PDGFR alpha)	ZLYTE	91
PDGFRA D842V	ZLYTE	98
PDGFRA T674I	ZLYTE	97
PDGFRA V561D	ZLYTE	97
PDGFRB (PDGFR beta)	ZLYTE	76
PDK1	ZLYTE	77
PDK1 Direct	ZLYTE	103
PEAK1	ZLYTE	91
PHKG1	ZLYTE	100
PHKG2	ZLYTE	99
PI4K2A (PI4K2 alpha)	Adapta	4
PI4K2B (PI4K2 beta)	Adapta	-1
PI4KA (PI4K alpha)	Adapta	7
PI4KB (PI4K beta)	Adapta	10
PIK3C2A (PI3K-C2 alpha)	Adapta	4
PIK3C2B (PI3K-C2 beta)	Adapta	9
PIK3C2G (PI3K-C2 gamma)	Adapta	2
PIK3C3 (hVPS34)	Adapta	-3
PIK3CA E542K/PIK3R1 (p110 alpha E542K/p85 alpha)	Adapta	13
PIK3CA E545K/PIK3R1 (p110 alpha E545K/p85 alpha)	Adapta	-3
PIK3CA/PIK3R1 (p110 alpha/p85 alpha)	Adapta	11
PIK3CA/PIK3R3 (p110 alpha/p55 gamma)	Adapta	4
PIK3CB/PIK3R1 (p110 beta/p85 alpha)	Adapta	-5
PIK3CB/PIK3R2 (p110 beta/p85 beta)	Adapta	-10
PIK3CD/PIK3R1 (p110 delta/p85 alpha)	Adapta	20
PIK3CG (p110 gamma)	Adapta	8
PIM1	ZLYTE	42
PIM2	ZLYTE	8
PIM3	ZLYTE	2
PIP4K2A	Adapta	1
PIP5K1A	Adapta	16

Kinase	Technology	% Inhibition Avg
PIP5K1B	Adapta	32
PIP5K1C	Adapta	7
PKMYT1	LanthaScreen	-61
PKN1 (PRK1)	ZLYTE	72
PKN2 (PRK2)	LanthaScreen	65
PLK1	ZLYTE	16
PLK2	ZLYTE	35
PLK3	ZLYTE	-10
PLK4	LanthaScreen	61
PRKACA (PKA)	ZLYTE	76
PRKACB (PRKAC beta)	LanthaScreen	48
PRKACG (PRKAC gamma)	LanthaScreen	18
PRKCA (PKC alpha)	ZLYTE	61
PRKCB1 (PKC beta I)	ZLYTE	19
PRKCB2 (PKC beta II)	ZLYTE	71
PRKCD (PKC delta)	ZLYTE	25
PRKCE (PKC epsilon)	ZLYTE	10
PRKCG (PKC gamma)	ZLYTE	81
PRKCH (PKC eta)	ZLYTE	-2
PRKCI (PKC iota)	ZLYTE	7
PRKCN (PKD3)	ZLYTE	95
PRKCQ (PKC theta)	ZLYTE	32
PRKCZ (PKC zeta)	ZLYTE	9
PRKD1 (PKC mu)	ZLYTE	97
PRKD2 (PKD2)	ZLYTE	100
PRKG1	ZLYTE	77
PRKG2 (PKG2)	ZLYTE	73
PRKX	ZLYTE	96
PTK2 (FAK)	ZLYTE	95
PTK2B (FAK2)	ZLYTE	96
PTK6 (Brk)	ZLYTE	71
RAF1 (cRAF) Y340D Y341D	LanthaScreen	0
RAF1 (cRAF) Y340D Y341D	ZLYTE	74
RET	ZLYTE	101
RET A883F	ZLYTE	99
RET G691S	LanthaScreen	-30
RET M918T	LanthaScreen	-40
RET S891A	ZLYTE	97
RET V804E	ZLYTE	94
RET V804L	ZLYTE	100
RET V804M	LanthaScreen	-5
RET Y791F	ZLYTE	100
RIPK2	LanthaScreen	77
RIPK3	LanthaScreen	18
ROCK1	ZLYTE	87
ROCK2	ZLYTE	83
ROS1	ZLYTE	98
RPS6KA1 (RSK1)	ZLYTE	94
RPS6KA2 (RSK3)	ZLYTE	96
RPS6KA3 (RSK2)	ZLYTE	95
RPS6KA4 (MSK2)	ZLYTE	75
RPS6KA5 (MSK1)	ZLYTE	49
RPS6KA6 (RSK4)	ZLYTE	97
RPS6KB1 (p70S6K)	ZLYTE	96
RPS6KB2 (p70S6Kb)	ZLYTE	37
SBK1	ZLYTE	81
SGK (SGK1)	ZLYTE	98
SGK2	ZLYTE	95
SGKL (SGK3)	ZLYTE	52
SIK1	LanthaScreen	82
SIK3	LanthaScreen	-1
SLK	LanthaScreen	88
SNF1LK2	ZLYTE	97
SPHK1	Adapta	17
SPHK2	Adapta	7
SRC	ZLYTE	89
SRC N1	ZLYTE	96
SRMS (Srm)	ZLYTE	63
SRPK1	ZLYTE	31
SRPK2	ZLYTE	32
STK16 (PKL12)	LanthaScreen	83
STK17A (DRAK1)	LanthaScreen	85
STK17B (DRAK2)	LanthaScreen	58
STK22B (TSSK2)	ZLYTE	74
STK22D (TSSK1)	ZLYTE	99

Kinase	Technology	% Inhibition Avg
STK23 (MSSK1)	ZLYTE	59
STK24 (MST3)	ZLYTE	85
STK25 (YSK1)	ZLYTE	45
STK3 (MST2)	ZLYTE	77
STK32B (YANK2)	LanthaScreen	61
STK32C (YANK3)	LanthaScreen	62
STK33	LanthaScreen	81
STK38 (NDR)	LanthaScreen	85
STK38L (NDR2)	LanthaScreen	59
STK39 (STLK3)	LanthaScreen	38
STK4 (MST1)	ZLYTE	93
SYK	ZLYTE	98
TAOK1	LanthaScreen	82
TAOK2 (TAO1)	ZLYTE	87
TAOK3 (JIK)	LanthaScreen	25
TBK1	ZLYTE	99
TEC	LanthaScreen	-9
TEK (Tie2)	ZLYTE	99
TEK (TIE2) R849W	LanthaScreen	-5
TEK (TIE2) Y1108F	LanthaScreen	72
TEK (TIE2) Y897S	ZLYTE	80
TESK1	LanthaScreen	37

Kinase	Technology	% Inhibition Avg
TGFBR1 (ALK5)	LanthaScreen	85
TGFBR2	LanthaScreen	77
TLK1	LanthaScreen	4
TLK2	LanthaScreen	33
TNIK	LanthaScreen	15
TNK1	ZLYTE	94
TNK2 (ACK)	LanthaScreen	71
TTK	LanthaScreen	54
TXK	ZLYTE	94
TYK2	ZLYTE	99
TYRO3 (RSE)	ZLYTE	92
ULK1	LanthaScreen	80
ULK2	LanthaScreen	56
ULK3	LanthaScreen	5
VRK2	LanthaScreen	32
WEE1	LanthaScreen	75
WNK1	LanthaScreen	2
WNK2	LanthaScreen	1
WNK3	LanthaScreen	7
YES1	ZLYTE	95
ZAK	LanthaScreen	77
ZAP70	ZLYTE	24

*Technology:
ZLYTE® - Biochemical assay - basd on differential sensitivity of phosphorylated and non-phosphorylated peptides to proteolytic cleavage (Thermo Fisher Scientific, USA)
Adapta® - Universal Kinase Assay - fluorescence-based immunoassay for the detection of ADP (Thermo Fisher Scientific, USA)
LanthaScreen® Binding - EU kinase binding assay (Thermo Fisher Scientific, USA)
