# Supplementary material for: Evaluation of Protein Kinase Inhibitors with PLK4 Cross-Over Potential in a Pre-Clinical Model of Cancer
Source: Int J Mol Sci. 2019 Apr 29;20(9):2112. doi: 10.3390/ijms20092112 (PMC6540285; doi:10.3390/ijms20092112)
Supplement: Supplementary file 1 [file ijms-20-02112-s001.zip › Supplementary Figure and Table/Supplementary Figure 1.pdf]

A

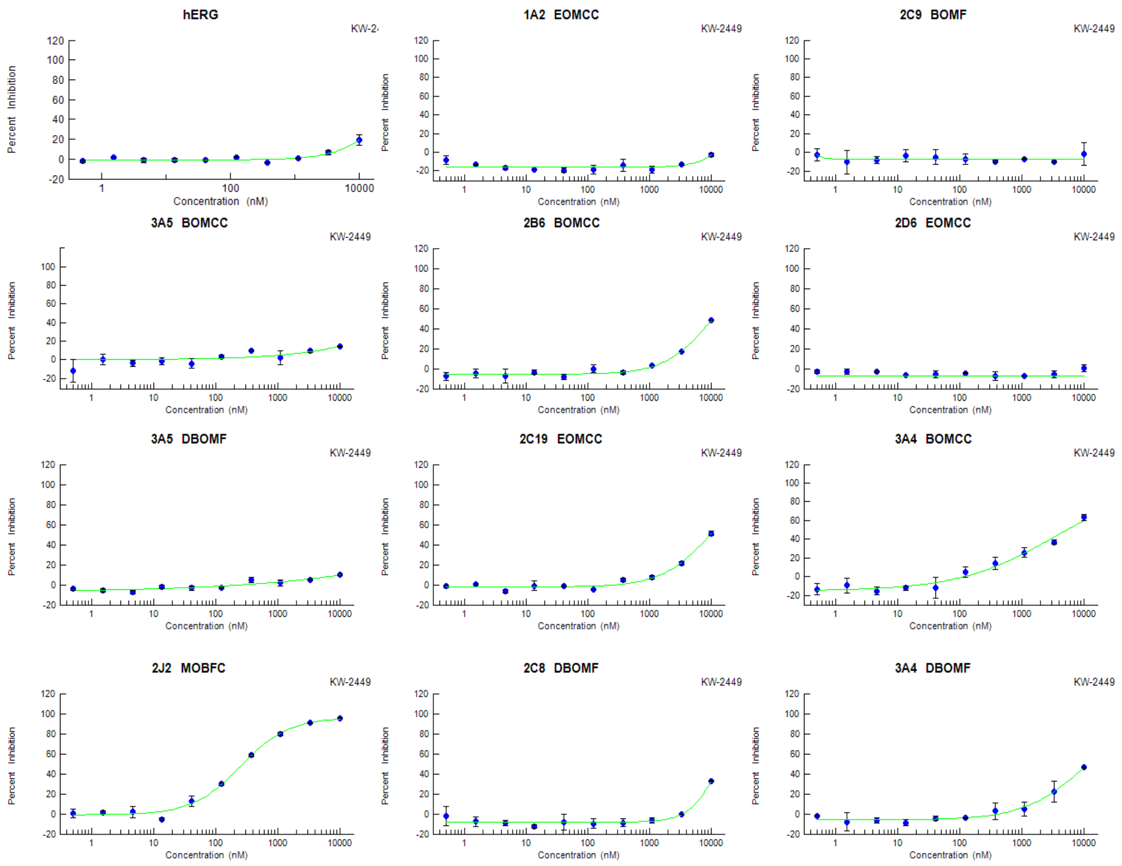

B

KW-2449 (10000nM)	% Inhibition
hERG	19
2J2 MOBFC - P450	96
3A4 BOMCC - P450	63
2C19 EOMCC - P450	51
2B6 BOMCC - P450	48
3A4 DBOMF - P450	46
2C8 DBOMF - P450	33
3A5 BOMCC - P450	14
3A5 DBOMF - P450	10
2D6 EOMCC - P450	1
2C9 BOMF - P450	-2
1A2 EOMCC - P450	-3

Supplementary Figure 1. KW-2449 – Drug safety and toxicology (SelectScreen P450 Vivid Biochemical Assay, Life Technologies, USA). (A) KW-2449 showed $IC_{50} \geq 10,000nM$ (the highest concentration tested) in screens, with positive controls, for each isoform considered of potential risk, except for 2B6 BOMCC (IC_{50} = 9,800nM), 2C19 EOMCC (IC_{50} = 9,410nM), 3A4 BOMCC (IC_{50} = 3,300nM) and 2J2 MOBFC (IC_{50} = 249). (B) Table of amount of inhibition KW-2449 has on enzymes implicit in drug metabolism.
